# RGD-p21Ras-scFv expressed prokaryotically on a pilot scale inhibits ras-driven colorectal cancer growth by blocking p21Ras-GTP

**DOI:** 10.1186/s12885-023-11686-5

**Published:** 2024-01-12

**Authors:** Peng Lin, Jing Qian, Cheng-Cheng Huang, Wen-Mang Xu, Yuan-Yuan Wang, Zi-Ran Gao, Shi-Qi Zheng, Peng Wang, Da-Qi Jia, Qiang Feng, Ju-Lun Yang

**Affiliations:** 1 Medical school, Kunming University of Science and Technology, Kunming, 650500 P.R. China; 2 Department of Pathology, 920th Hospital of the Joint Logistics Support Force of PLA, 212 Daguan Rd, Kunming, 650032 P.R. China; 3 Faculty of Life science and Technology, Kunming University of Science and Technology, Kunming, 650500 P.R. China; 4 The Graduate School, Kunming Medical University, Kunming, 650500 P.R. China

**Keywords:** Pilot scale, scFv, GTP, Colorectal cancer, Signaling pathway

## Abstract

**Background:**

Ras gene mutation and/or overexpression are drivers in the progression of cancers, including colorectal cancer. Blocking the Ras signaling has become a significant strategy for cancer therapy. Previously, we constructed a recombinant scFv, RGD-p21Ras-scFv by linking RGD membrane-penetrating peptide gene with the anti-p21Ras scFv gene. Here, we expressed prokaryotically RGD-p21Ras-scFv on a pilot scale, then investigated the anti-tumor effect and the mechanism of blocking Ras signaling.

**Methods:**

The *E. coli* bacteria which could highly express RGD-p21Ras-scFv was screened and grown in 100 L fermentation tank to produce RGD-p21Ras-scFv on optimized induced expression conditions. The scFv was purified from *E. coli* bacteria using His Ni-NTA column. ELISA was adopted to test the immunoreactivity of RGD-p21Ras-scFv against p21Ras proteins, and the IC50 of RGD-p21Ras-scFv was analyzed by CCK-8. Immunofluorescence colocalization and pull-down assays were used to determine the localization and binding between RGD-p21Ras-scFv and p21Ras. The interaction forces between RGD-p21Ras-scFv and p21Ras after binding were analyzed by molecular docking, and the stability after binding was determined by molecular dynamics simulations. p21Ras-GTP interaction was detected by Ras pull-down. Changes in the MEK-ERK /PI3K-AKT signaling paths downstream of Ras were detected by WB assays. The anti-tumor activity of RGD-p21Ras-scFv was investigated by nude mouse xenograft models.

**Results:**

The technique of RGD-p21Ras-scFv expression on a pilot scale was established. The wet weight of the harvested bacteria was 31.064 g/L, and 31.6 mg RGD-p21Ras-scFv was obtained from 1 L of bacterial medium. The purity of the recombinant antibody was above 85%, we found that the prepared on a pilot scale RGD-p21Ras-scFv could penetrate the cell membrane of colon cancer cells and bind to p21Ras, then led to reduce of p21Ras-GTP (active p21Ras). The phosphorylation of downstream effectors MEK-ERK /PI3K-AKT was downregulated. In vivo antitumor activity assays showed that the RGD-p21Ras-scFv inhibited the proliferation of colorectal cancer cell lines.

**Conclusion:**

RGD-p21Ras-scFv prokaryotic expressed on pilot-scale could inhibited Ras-driven colorectal cancer growth by partially blocking p21Ras-GTP and might be able to be a hidden therapeutic antibody for treating RAS-driven tumors.

**Supplementary Information:**

The online version contains supplementary material available at 10.1186/s12885-023-11686-5.

## Introduction

*ras* gene is the first oncogene isolated from human bladder cancer cells [1]. The ras gene has three main subtypes, namely N-ras, H-ras, and K-ras, encoding four Ras proteins: N-Ras, H-Ras, K-Ras4A, and K-Ras4B, respectively. Among them, K-Ras4A and K-Ras4B are different splicing forms of K-Ras protein. These proteins all exist in two forms: the activated state bound to GTP (Ras GTP) and the inactivated state bound to GDP (Ras-GDP). Ras protein(p21Ras) regulates cell differentiation, proliferation, and survival through the “on/off” cycle of activated Ras GTP and deactivated Ras GDP. Under physiological conditions, this state change is balanced. Once the balance is lost, such as point mutations at codons 12, 13, or 61, the hydrolysis rate of GTP in Ras GTP will decrease, meaning that Ras protein continues to exist in the activated state as Ras-GTP style, which will promote cell proliferation and lead to the occurrence of cancer [2]. Research has found that about 30% of human tumors harboured ras gene mutations [3]. Every year, more than 3 million cancer patients worldwide are caused by ras mutations. Such as pancreatic cancer, colorectal cancer, lung cancer, peritoneal cancer, bladder cancer cancer, bile duct cancer and melanoma [4]. In recent years it was found that overexpression of wild type p21Ras could lead occurence of cancer [5]. Therefore, p21Ras is a potential therapeutic target for *ras* gene driven tumors, and the development of drugs against p21Ras is of great significance for the diagnosis and treatment of ras gene driven tumors.

The drugs development for the p21Ras itself are currently mainly focused on two directions: small molecule inhibitors and therapeutic antibodies. At present, a few small molecule inhibitors have entered the clinical trial stage, and Sotorasib (AMG510) and Adagrasib (MRTX849) are approved for clinical practise [7–9]. However, anti-p21Ras antibody drugs have not yet been available.

As early as the 1980s, anti-p21Ras antibodies, Y13-259 and RASK1-16 etc. were reported, but they could not penetrate cell to bind p21Ras within cytoplasm [10, 11]. Previously, we prepared anti-p21Ras single chain antibodies(p21Ras-scFv) based on phage display library technology [12]. We inserted the p21Ras scFv gene into the adenovirus vector and infected tumor cells, achieving intracellular expression of the p21Ras-scFv. In vitro and in vivo experiments have shown that the anti-p21Ras-scFv has unexpected anti-tumor activity [13–19].

In order to gain anti-p21Ras-scFv which can penetrate tumor cell, we linked the RGD transmembrane peptide gene to the p21Ras-scFv gene and expressed the RGD-p21Ras-scFv through a prokaryotic expression system. In vitro experiments have shown that it can enter tumor cells and has anticipated anti-tumor activity [20–22]. However, the above study obtained recombinant antibodies through small-scale expression in laboratory shaking bottles. To convert recombinant antibodies into clinical anti-tumor drugs, it is necessary to explore the conditions for large-scale preparation and determine that the recombinant antibodies prepared on a large scale have the same immunoreactivity and anti-tumor activity. Therefore, this study established a pilot scale expression process to prepare “gram level” RGD-p21Ras-scFv protein, and verified its immunoreactivity and anti-tumor activity. The purpose of this study is to explore the possibility of further industrial development of the recombinant antibody, laying the foundation for the commercial production of RGD-p21Ras-scFv protein.

## Materials and methods

### Cell cultures and reagents

The Kunming Cell Bank of the Chinese Academy of Sciences, including human cell lines CCD841, A549, AGS, AsPC-1, HePG-2, MDB-MB-231, MIApaca-1, PANC-1, U251, HT29, SW837, LS180, SW480, and HCT116 all cells; and human embryonic kidney cell line 293T. The growth of cells was made in the corresponding medium (DMEM: SW480, SW837, 293T; RPMI 1640: HCT116, HT29; MEM EAGLE: LS180) added with 10% fetal bovine serum (Excell Bio) and 1% penicillin/streptomycin (Basic Medium) at 37 °C in a 5% carbon dioxide incubator. The KRAS (G12C) inhibitor was dissolved in DMSO.

### Construction of expression plasmid

The gene encoding RGD-p21Ras-scFv was obtained by PCR amplification using the paired primer with the RGD-p21Ras-scFv in the pet28a plasmid (preserved at our laboratory) as a template [20]. The primers were synthesized by TsingKe Biological Technology Inc. (Kunming, China), and PCR products were subcloned into the pClone 7 plasmid between Hind III and Kpn I and transfected into DH5α strain. Then, the pClone 7 plasmid and the PET-28a (+) were digested with Hind III and NdeI, the pClone 7 plasmid and the PET-32a (+) were digested with Hind III and KpnI, the pClone 7 plasmid and the PET-22b were digested with NdeI and Hind III to acquired fusion gene. The fragments of RGD-p21Ras-scFv were recovered from the agarose gel(Promega), purified, and ligated by T4 DNA ligase, so the RGD-p21Ras-scFv gene was inserted into different expression vectors and then transfected into DH5α according to the standard protocol [23]. Using the PCR and DNA sequencing to identify the RGD-p21Ras-scFv gene fragment insert in the correct position.

The research adopted the primer pairs as below: 5’-CCTCCCAGCTCTGGTATTGC-3’(Forward) and 5’-AGGGTTCTGTGAGTTTGATT-3’(Reverse) for T7 sequence using in plcone7 plasmid identification. The 5’-CATATGGCATGTGATTGTCG-3’(Forward) and 5’-AAGCTTTTATCACCGTTTGA-3’(Reverse) for RGD-p21Ras-scFv sequence using in expression plasmid identification. The PCR reaction program was: 94 °C, 5 min; 94 °C, 50s; 55 °C, 1 min; 72 °C, 45 S; 72 °C, 10 min; 32 cycles.

### Selection and optimization of expression condition

The expression vector was transformed into E.coil Origami(DE3), Origami B (DE3) and BL21(DE3). After recovery, 200µL of the cells were spread on a LB solid medium (1% peptone, 1% yeast extract, and 0.5% sodium chloride, 1.5% agar, pH 7.0) a) containing 100 µg /ml ampicillin and 50 µg/ml Kanamycin. The plate was upside down and incubated at 37℃ overnight. The inserted RGD-p21Ras-scFv gene was confirmed by PCR and DNA sequencing. Identified single clones were placed in 5ml LB medium(1% peptone, 1% yeast extract, and 0.5% sodium chloride, pH 7.0) containing 100 µg /ml ampicillin and 50 µg/ml Kanamycin shaking at 37℃, 200 rpm overnight.To optimize the highest level of RGD-p21Ras-scFv recombinant antibody expression, the comparison between the different strains, different cultures (LB and Isopropyl-β-Dthiogalactopyranoside, ZYM5052), and expression form in soluble or inclusion body were evaluated. Briefly, the isopropyl-β-Dthiogalactopyranoside (IPTG) induced methods: The bacteria were inoculated into a 200ml shake flask containing 30ml LB containing 100 µg /ml ampicillin and 50 µg/ml Kanamycin, culturing with proportion of bacteria involved was at ratio of 1/100 and incubated at 37 °C, 200 rpm in a shaker until the OD value (at 600 nm) reached 0.6–0.8. At this time point, different concentration of IPTG from 0.2mM to 1.6mM was added to the LB culture followed by shaking at 37 °C, and 200 rpm for 5 h, respectively. Then, after the most suitable concentration of IPTG was chosen, the induced time was from 4 to 20 h to be selected.

The self-inducing methods: This method was using ZYM-5052 culture which contains 1% Tryptone, 0.5% Yeast extract, 25mM Na_2_HPO_4_, 25mM KH2PO4, 50mM NH_4_Cl, 5 m MNa_2_SO_4_, 0.5% Glycerol, 0.05% Glucose, 0.2% α-lactose, 2 m Mg_2_SO_4_, 0.2x Trace elements [24]. The bacteria were inoculated into a 200ml shake flask containing 30ml LB containing 100 µg /ml ampicillin and 50 µg/ml Kanamycin and culture with the proportion of bacteria involved was at a ratio of 1/100 at 37 °C, 200 rpm shaking until the OD value reached 0.6–0.8, then reducing the temperature to 20 °C with 200 rpm shaking 20 h. Recombinant bacteria was harvested by centrifugation, and lysed by sonication into the buffer. The supernatants were harvested by centrifugation at 12,000 rpm for 30 min at 4 °C. Both soluble and insoluble bodies were analyzed by 12% sodium dodecyl sulfate-polyacrylamide gel electrophoresis (SDS-PAGE).

### Recombinant protein expression and pilot-scale fermentation

Small-scale expression in the laboratory: The bacteria were inoculated into a 1 L shake flask containing 500ml LB containing 100 µg /ml ampicillin and 50 µg/ml Kanamycin and cultured under the condition of self-inducing method.

Pilot-scale fermentation: Before inoculation in bioreactors, the seed strain cultures were prepared in a total volume of 1 L. Briefly, inoculating 1ml *E. Coli* Orignami (DE3) cells into a 1 L baffled shake flask containing 400 mL of LB medium with 100 µg /ml ampicillin and 50 µg/ml Kanamycin 200 rpm shaking until the OD (600 nm) value reached 0.6-0.8 nm. The seed culture was transferred to a 100 L bioreactor containing 60 L of ZYM-5052 medium with lactose doubled and the rest unchanged. The temperature and stirring were started at 37 °C and 200 rpm, respectively. When the OD (600 nm)value reaches 0.6–0.8, the temperature drops to 20℃ continuing cultures for 16–18 h. The pH was maintained at 7.0-7.1 by the addition of 30% H_3_PO_4_ and NaOH. The concentration of dissolved oxygen was maintained at 30-35% of air saturation. When the DO (dissolved oxygen) level could not be maintained by increasing oxygen, the feed rate was controlled by adding medium (yeast 40 g/L, peptone 80 g/L, MgSO_4_·7H_2_O 10 g/L, lactose 100 g/L). A DO control feed approach was used to control the feeding rate. After fermentation, pellets were harvested by using continuous flow centrifuges centrifugation at 12,000 ×g, 4 °C, and the pellet was frozen at − 70 °C.

### RGD-p21Ras-scFv recombinant antibody purification, refolding

The RGD-p21Ras-scFv was purified by affinity chromatography and ion exchange chromatography. After fermentation, the cells were harvested. The 31 g cell pellets were ultrasonically disrupted and centrifuged as 12,000 g for 20 min. The inclusion bodies were dissolved with binding buffer, they were filtered using a 0.45 nm filter membrane and following purification. Firstly, the recombinant protein purification with Ni Sepharose 6 Fast Flow (GE Healthcare,) under the denaturation condition. The Ni-NTA resin was washed with binding buffer I (20 mM phosphate, containing 10 mM imidazole and 500 mM NaCl, 8 M urea, and pH 7.4) until OD280 of effluent reached the baseline. Contaminating proteins were eluted from the column with wash buffer II l containing (20 mM phosphate,20 mM imidazole 500 mM NaCl, 8 M urea, and pH 7.4). Finally, the protein was eluted with elution buffer (20 mM phosphate,250 mM imidazole, 500 mM NaCl, 8 M urea, and pH 7.4). Then, the proteins were refolded under the refolding condition as the prior description [20]. By using a urea gradient for refolding, the dialysis sequence starts with reversion solution I for 6 h with 6 M urea. Dialysis refolding is performed at low temperature using a magnetic stirrer, and dialysis refolding is completed when dialysis reversion solution IV is ready with 0 M urea, and finally, RGD-p21Ras-scFv recombinant antibody is dialyzed in 0.01 M PBS (containing 10% glycerol) buffer. In the last, the DEAE Sepharose FF column chromatography purification was performed. The eluted with buffer containing different concentrations of NaCl (0.3 mol/L, pH 8.0). The purity of fusion protein was assessed using SDS-PAGE, and its concentration was evaluated with the BCA Protein Assay Kit.

### ELISA to identify RGD-p21Ras-scFv immunoreactivity

Respectively, dilute K-p21RAS, N-p21RAS and H-p21RAS antigens to a final concentration of 5ug/ml using pH 9.6 substrate buffer, incubation at 4 °C overnight; add 1% BSA-PBS for closure, incubate at 37 °C for 1 h and wash with TBST; respectively, add RGD-p21Ras-scFv recombinant antibodies at 1:50, 1:100, 1:200, 1:400, 1:800, 1:1600, 1:3200 and conduct 1-hour incubation at 37 °C and wash by TBST; add diluted anti-His tag antibody, make 1-hour incubation at 37 °C and washed; add diluted anti-His tag antibody(1:4000), make 1-hour incubation at 37 °C and washed; add diluted secondary antibody(1:200) of enzyme standard, incubate at 37 °C for 1 h and perform TMB color development. An enzyme marker was adoptedto measure the absorbance value at a wavelength of 450 nm.

### Counting Kit-8 assay to determine IC50

After being seeded into 96-well culture plates with 5000 cells per well, HT29, SW837, LS180, SW480 and HCT116 cells were treated with different doses (0 μm, 2.5 μm, 5 μm, 10 μm, 20 μm, 40 μm, 80 and 160 μm)) of the drug for 48 h. Cell viability was checked with the Cell Counting Kit-8 assay (CCK8, APExBIO, Lot: K101816122EF5E) based on the manufacturer’s protocol, and IC50 values were calculated.

### SDS-PAGE

Add 5×loading buffer with protein samples to make the final concentration of buffer to 1X. mix well using a vortex shaker before loading, and microcentrifuge in a centrifuge, heat the EP tubes in a preheated metal bath at 98 °C for 10 min. load the samples separately according to the experimental order, first run 85 V to the separation gel and then adjust the voltage to 110 V. after electrophoresis, put the separation gel into Komas Brilliant Blue After staining for half an hour, the separation gel was decolorized by boiling with distilled water until clear protein bands could be observed, stopped decolorization and photographed and recorded.

### Immunofluorescence colocalization

HT29, SW837, LS180, SW480 and HCT116 cells were inoculated at approximately 50% density on microscope slides and allowed to adhere overnight. The cells were co-cultured with 30 µM RGD-p21Ras-scFv for 5 h. The 30-minute fixation of cells was made with 4% paraformaldehyde, followed by washing with PBS; the 10-minute permeabilization of cells was made with 0.2% Triton X-100. After 1-hour incubation with primary anti-Pan-Ras mouse monoclonal antibody (sc-166,691, Santa Cruz) and His-tag rabbit monoclonal antibody (#12,698, CST) at 37 °C, the 1-hour incubation of secondary anti-Goat anti-mouse IgG/TRITC (1: 200, 113,608, ZSGB-BIO) and Goat anti-rabbit IgG/FITC (1 :200, 135,850, ZSGB-BIO) was made at 37 °C in the dark. Then, 7 µl of DAPI (Solarbio, C0065) was put to every slide, and the slides were sealed and protected from light. Images were taken with a fluorescent camera (OLYMPUS, BX51).

### Pull-down assay

The RGD-p21Ras-scFv with FLAG tag, wild-type p21Ras gene with MYC tag and p21Ras^G12C^ gene mutation with MYC tag plasmids were synthesized by Shanghai Jikai Gene Medical Technology Co., Ltd. Single colonies were selected by the plate scribing method, inoculated into liquid medium containing antibiotics, and shaken at 220 rpm at 37 ℃ for 12–16 h. Finally, the extraction of plasmid was made based on the standard experimental step using the EndoFree Mini Plasmid Kit II (Cat. #DP118-02) from TIANGEN BIOTECH (Beijing) Co., Ltd., and stored at -20 °C until use. Finally, using a Lipofectamine™ 3000 Transfection Kit (Thermo, 2,413,975, USA), the plasmid was transfected into 293T cells. The RGD-p21Ras-scFv plasmid with FLAG and the wild-type p21Ras plasmid with MYC were transfected into 293T cells. RGD-p21Ras-scFv with FLAG and p21Ras^G12C^ with Myc were grouped, and blank plasmids with FLAG and RGD-p21Ras-scFv with FLAG were transfected into 293T cells (human embryonic kidney cells) for 36 h and then lysed in lysis buffer. A FLAG-labeled protein immunoprecipitation kit (magnetic bead method) and BeyoMag™ Anti-Flag Magnetic Beads (Beyotime, P2181S, P2118-2ml) were used, and 500 µl protein samples were incubated with 20 µl magnetic bead suspension at 4 °C overnight, magnetically separated for 10 s and washed. Finally, the protein samples obtained from these samples were eluted by the 3X FLAG competitive elution method. Proteins were detected by western blotting with an anti-FLAG tag mouse monoclonal antibody (1:1000, Proteintech, 66008-4-lg), anti-MYC tag mouse monoclonal antibody (1:1000, Proteintech, 60003-2-lg) and the appropriate goat anti-mouse IgG/HRP secondary antibody (1:10000, ZSGB-BIO, ZB-5305).

### Molecular Docking

Build the RGD-p21Ras-scFv model using Piper (Clus Pro 2.0), save the PDB file. Download the PDB files (K-p21Ras, ID: 4LDJ; H-p21Ras, ID: 6E6P; N-p21Ras, ID: 3CON) at https://www.rcsb.org/. Open the antibody docking mode of the Piper for antigen-antibody docking. Import the pdb of K-p21Ras; H-p21Ras; N-p21Ras; in separate steps for optimization (hydrogen addition, charge calculation, etc.). Import RGD-p21Ras-scFv.pdb. Close the non-CDR regions. Fourier-transformed surface fit search using CDR regions with antigens (Note: find the most appropriate antigen-antibody docking model among 70,000 conformations combined with the actual situation) [25].

### Molecular dynamics simulation

Gromacs (version 2022.3) software [26, 27] was adopted for the molecular dynamics simulation. First, the molecularly docked pdb was imported and the pdb was converted to the protein format of Gromacs, and the amber99sb-ildn force field and the tip3p water model were specified. Then, add the periodic bounding box shaped as a vertical square at a distance of 1.2 nm from the protein, and add the just-set tip3p water in the box after the following two lines are added Na ions to neutralize the charge in the system to make it electrically neutral. After performing the energy minimization process the system is subjected to NVT equilibrium and NPT equilibrium. Finally, start the simulation.

### Western blotting

After different doses (described above) of drug treatment for 48 h, cells (HT29, SW837, LS180, SW480 and HCT116) were lysed in RIPA lysis and extraction buffer (Cat# 89,900, Thermo, USA). BCA protein assay kits (Beyotime, China) were used for collection and quantification of proteins. Electrophoresed by 10% SDS‒PAGE, proteins were moved to a PVDF membrane (Bio-Rad, USA) with transfer buffer. The 1-hour blocking of membranes was made with 5% nonfat milk powder, followed by incubation with primary antibodies (1:1000) against β-actin (Cell Signaling Technology (CST), #3700), PI3K (Proteintech, 60225-1-Ig), P-PI3K (CST, #17,366), ERK1/2 (Proteintech, 67170-1-Ig); P-P44/42MAPK (ERK1/2) (CST, #4370), AKT (CST, #4691), P-AKT (CST, #4060), MEK1/2 (Proteintech, 110499-1-AP), and P-MEK1/2 (CST, 9154) overnight at 4 °C. The next day, the 1-hour incubation of membranes was made with the appropriate secondary antibodies (goat anti-rabbit IgG/HRP (1:10000, ZSGB-BIO, ZB-2301) or goat anti-mouse IgG/HRP (1:10000, ZSGB-BIO, ZB-5305)) at room temperature. A western blotting detection system (Bio-Rad, USA) was adopted to visualize protein bands.

### Ras pull-down assay

Ras pull-down assays were made by the Active Ras pull-down kit (Thermo, 16,117). First, cells were lysed using lysis buffer to obtain the protein. Second, by 15-minute incubation of the supernatant of the total cell lysate with guanosine triphosphate labeled with S (GTPγS) on a gamma phosphate base at room temperature, the target protein (p21Ras-GTP, Total p21Ras) [28] was obtained. Finally, the protein samples were subject to resuspending using the agarose beads from the kit, washing with 400 µl of wash buffer, and 30-second centrifugal at 6000 g. Primary antibody anti-Ras antibody (1:250, Thermo) and secondary antibodies (goat anti-Rabbit IgG/HRP (1:10000, ZSGB-BIO, ZB-2301)) were adopted to make western blotting.

### Tumor xenograft animal experiments

The Laboratory Animal Ethics Committee of 920th Hospital, Yunnan, China approved animal experiments. Healthy BALB/c nude mice (male, N = 30, 5–6 weeks old, and weighing 15–18 g) were offered by China Hunan Slake Jingda Experimental Animal Co., Ltd. After being housed under specific pathogen-free (SPF) situations at 25 °C with 50% humidity, all animals can access to food and water freely. The inoculation of HT29 and SW48 cells (1 × 10^6^ cells/mouse) in 0.15 ml of serum-free DMEM was made into the left armpits of the nude mice. After 7 days, the tumor volume was 50–100 mm^3^ and the random division of mice was made into 5 groups (n = 6 for each group): RGD-p21Ras-scfv treatment group (150 µg/pc/2 days), PBS (150 µg/pc/2 days), RGD (150 µg/pc/2 days), DMSO (150 µg/pc/2 days), KRAS(G12C) inhibitor (150 µg/pc/2 days). The mouse body weight and the length (a) and width (b) of the tumor were supervised every two days with calipers. The calculation of tumor volume (V) was made below: V = ab^2^/2. The tumor tissues were collected, and the tumor was weighed after the mice were anesthetized with persistent isoflurane on Day 21.

### Hematoxylin and eosin staining and histology

After 21 days of treatment, the mice were killed. The heart, liver, spleen, lung, kidney, brain, intestine, stomach tissues, and a part of the tumor were isolated, fixed in 10% paraformaldehyde buffer for HE staining. The freezing of some tumors and normal tissues was made in liquid nitrogen for other research.

### Immunohistochemistry (IHC)

Paraffin sections of tumor tissue were stained immunohistochemically with anti-Ki67 antibody (ZSGB-BIO, 1:200, ZM-0166), anti-His-tag mouse monoclonal antibody (1:10000, Proteintech, 66005-1-lg), anti-LCK antibody (ZSGB-BIO, 1:200, ZM-0329), anti-villin antibody (ZSGB-BIO, 1:200, ZM-0261), and anti-vimentin antibody (ZSGB-BIO, 1:200, ZM-0261) followed by incubation with the secondary antibody and DAB color development. The number of Ki67-positive cells divided by the total number of resting cells [29] was used to calculate the proliferation index (PI).

### Statistical analysis

Data were shown as the mean ± SD of three repeated tests. The statistical significance was compared with Student’s t-test and one-way ANOVA by GraphPad Prism 8.0 (San Diego, CA, USA). *P < 0.05 were considered significant.

## Results

### Construction and identification of the recombinant expression plasmid

**Fig. 1:**
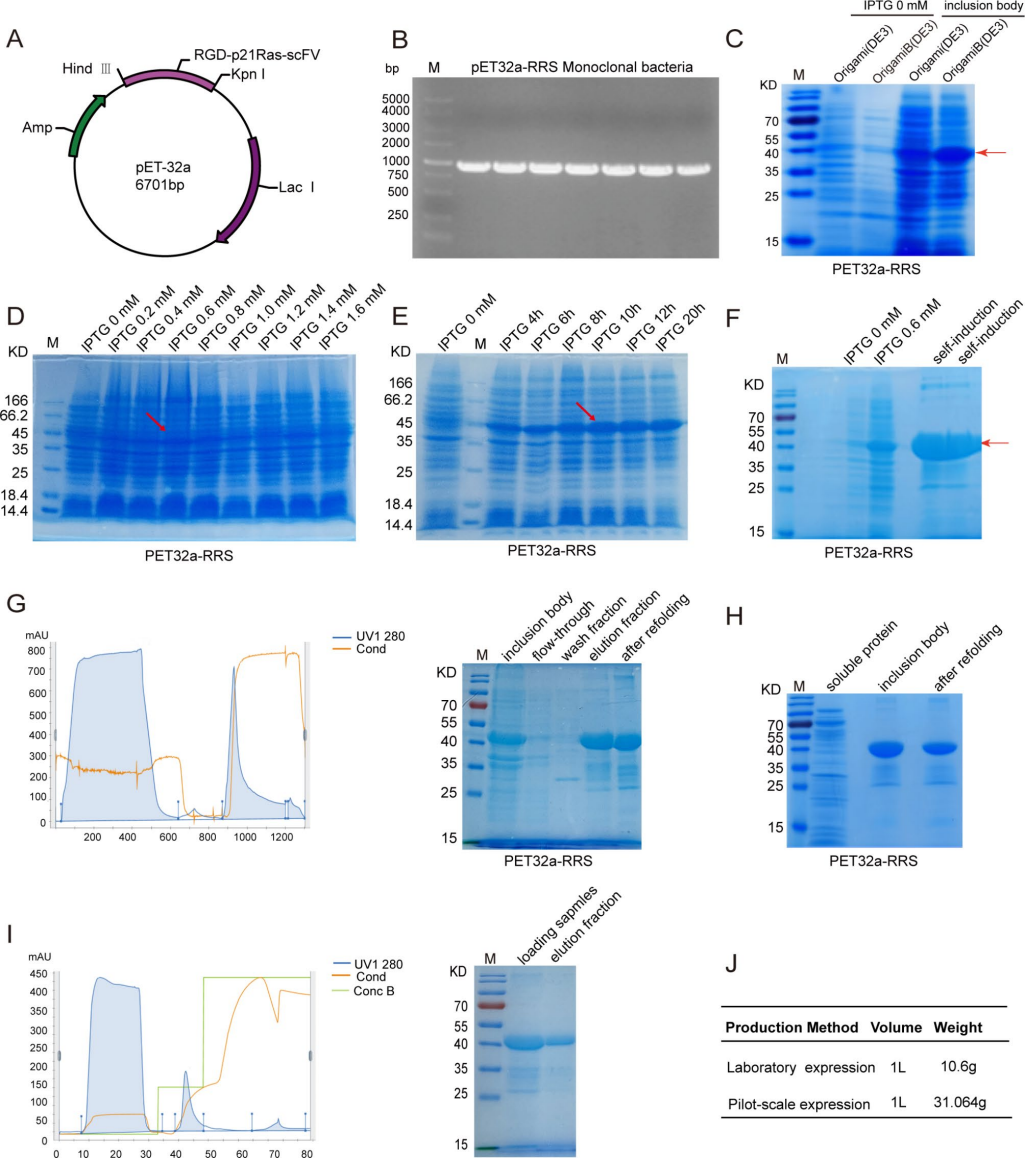
Expression and purification of RGD-p21Ras-scFv recombinant antibody in E.coil system. **(A)**The recombinant expression plasmids pET32a-RRS were set up by inserting the RGD-p21Ras-scFv gene into the corresponding enzyme cleavage site of the plasmid vector. **(B)** PCR showed the RGD-p21Ras-scFv gene was successfully transferred into pET32a plasmid. **(C)** The highest expression of RGD-p21Ras-scFv was detected in Origami(DE3) and Origami B(DE3) inclusion bodies by SDS-PAGE. The red arrow showed the target protein. **(D)** The RGD-p21Ras-scFv was expression in different concentration of IPTG from 0.2mM to 1.6mM. The red arrow shows the optimum inducible expression conditions. **(E)** After the suitable concentration of IPTG was chosen(0.6mM), the induced time was investigated from 4 to 20 h. The red arrow shows the optimum inducible expression conditions. **(F)** SDS-PAGE showed theRGD-p21Ras-scFv was more highly expressed in the self-induction system than in IPTG as detected by SDS-PAGE analysis. The red arrow shows the target protein. **(G)** Affinity chromatogram (left) and SDS-PAGE (right) of RGD-p21Ras-scFv recombinant antibody from Ni2 + affinity resin. The wash fraction contains 20 mM imidazole, and the elution fraction contains 250 mM imidazole. **(H)** RGD-p21Ras-scFv recombinant antibody was more highly expressed in inclusion bodies than in soluble proteins as detected by SDS-PAGE analysis. **(I)** Ion-exchange chromatography of RGD-p21Ras-scFv from DEAE resin (left) and SDS-PAGE (right). The refolding protein to loading up after adjusted the pH at 8.0 and the elution fraction with 0.3 mol/L NaCl in elution buffer. **(J)** Comparison of the wet weight of bacteria expressed in small scale shaker in laboratory and fermentation. Fermentation can significantly increase the yield (cell wet weight)

Through the standard procedure for molecular cloning, the RGD-p21Ras-scFv recombinant antibody gene fragment was double-digested from the plcone7 vector. The gene fragment was inserted into three expression plasmids pET32a, pET28a, and pET22b using T4 ligase, and named the recombinant plasmid pET32a-RRS (Fig. 1A), pET28a-RRS (Supplementary Fig. 1A), and pET22B-RRS (Supplementary Fig. 1B), respectively. The PCR and DNA sequencing also identified the gene fragments were all correctly and inserted correctly in the pET32a-RRS (Fig. 1B), pET28a-RRS (Supplementary Fig. 1C), pET22B-RRS (Supplementary Fig. 1D).

Expression and fermentation of RGD-p21Ras-scFv recombinant antibody.

Three expression plasmids were transferred into different expression bacteria BL21(DE3), Origami (DE3), and Origami B(DE3). At 37℃ and induced by 1mM IPTG for 5 h, the recombinant proteins expressed in soluble and inclusion body form were screened by SDS-PAGE. Among the three expressing bacteria, inclusion body expression was more than soluble expression (Fig. 1C, supplementary Fig. 1E-F), and most proteins are expressed in the form of inclusion bodies in Origami (DE3) (Fig. 1C). The molecular weight of the recombinant antibody was in the range of 35–45 kDa, which was consistent with the predicted results. To increase the expression level, we further screened the concentration and induction time of IPTG and the use of different media. The experimental results showed that the inclusion body expression was the highest at 37℃ and 0.6mMIPTG for 10 h of induction (Fig. 1D-E). Moreover, compared with the IPTG-induced expression of traditional LB medium and ZYM-5052 self-induced medium, we found that ZYM-5052 medium could express more inclusion bodies, much higher than that of IPTG induced expression (Fig. 1F).

After controlled fermentation by dissolved oxygen feedback and continuous feed feeding, the optimal production conditions were preliminarily determined, and 1863.84 g total of bacteria cell pellets could be obtained by 60 L fermentation, and the average yield was 31.064 g /L (Wet weight of bacteria). At the same time, most proteins are still expressed in the form of inclusion bodies. Compared with the laboratory wet weight of 10.6 g/L of bacteria, fermentation by the dissolved oxygen feedback method greatly increased the yield of bacteria (Fig. 1J).

### Two-step of purification of RGD-p21Ras-scFv recombinant antibody

After fermentation, 14.3 g inclusion bodies were obtained from the cell pellets by ultrasonic crushing of 31 g bacteria cell pellets. Under denaturation conditions, Ni-NTA fast flow affinity chromatography was performed first. Protein elution was performed with 250 mM imidazole. The target protein 286.7 mg was obtained from 14.3 g dissolved inclusion body protein solution by Ni column affinity chromatography. Then, urea gradient refolding is performed at low temperatures, and the refolding loss protein is about 3-7% of the total refolding protein (Fig. 1G). DEAE ion exchange chromatography was performed after renaturation. DEAE is a commonly used weak anion exchange filler whose adsorption affinity is affected by pH value. DEAE column chromatography results showed that most of the target proteins were eluted by 0.3 mol/L NaCl. The protein with low binding to the DEAE column had a loss of about 40% (Fig. 1I). In addition, soluble proteins were purified by Ni-NTA affinity chromatography. The results showed that more target proteins were obtained from inclusion bodies than from soluble proteins by affinity chromatography (Fig. 1H). SDS-PAGE identification showed that the target protein size was consistent. The results also showed that the purity of the obtained recombinant protein could reach more than 85%.

### RGD-p21Ras-scFv binds stably to the p21Ras protein and is structurally stable upon binding

**Fig. 2:**
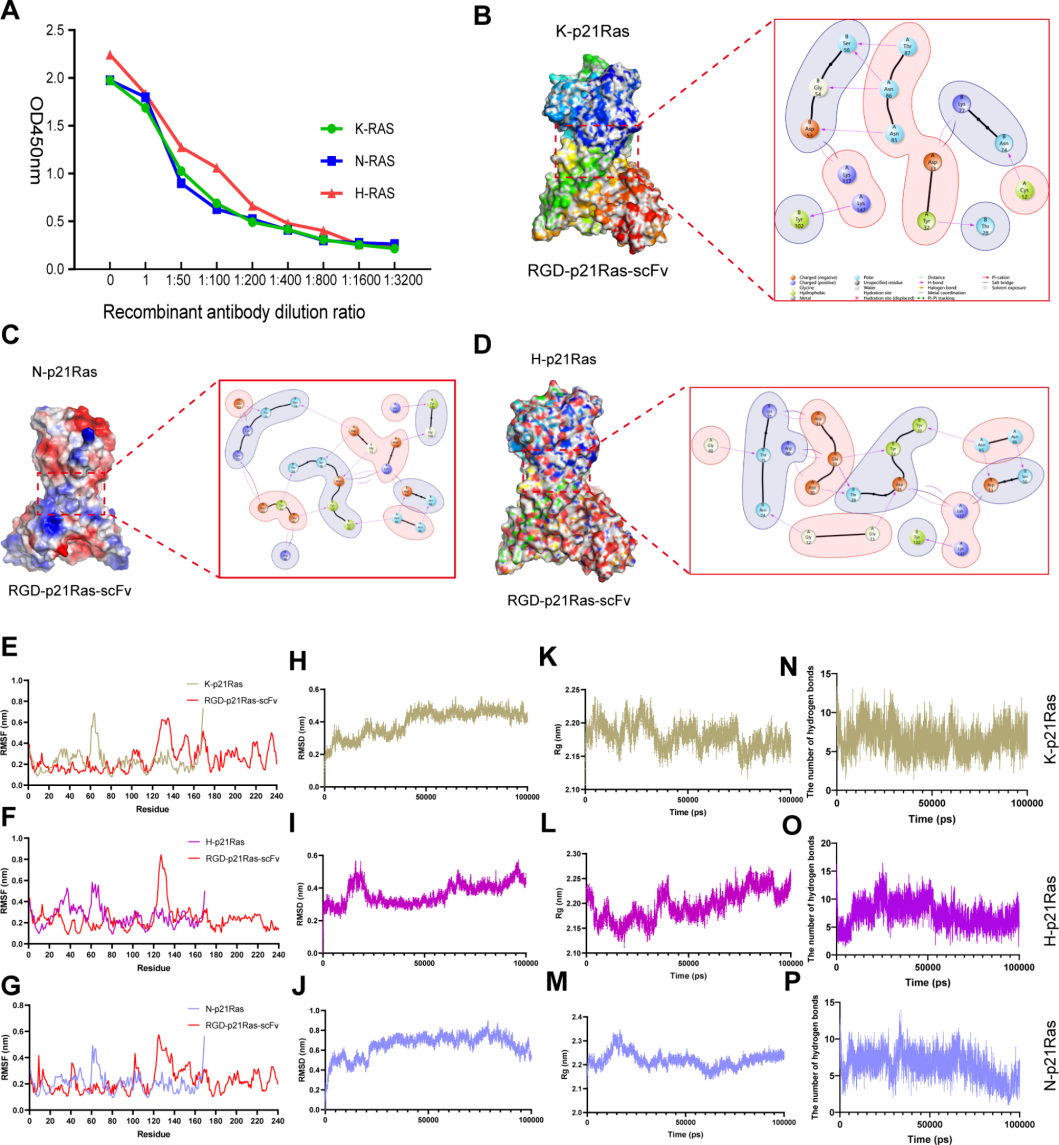
RGD-p21Ras-scFv could bind stably to K-p21Ras, H-p21Ras and N-p21Ras proteins. **(A)** RGD-p21Ras-scFv was immunoreactive with K-p21Ras, H-p21Ras and N-p21Ras at 1:800 by ELISA. **(B, C, D)** The molecular docking model of RGD-p21Ras-scFv to K-p21Ras, H-p21Ras and N-p21Ras proteins, The RGD-p21Ras-scFv binds to p21Ras through hydrogen bonding and salt bridges et al. **(E-P)** Molecular dynamics simulated the interaction and stability of RGD-p21Ras-scFv upon binding to p21Ras. The complexes formed by RGD-p21Ras-scFv with K-p21Ras, H-p21Ras and N-p21Ras fluctuated very little and were highly stable during the simulation process, and the graphs demonstrated RMSF; RMSD; Rg; and the number of hydrogen bonding from left to right, respectively

To determine the immunoreactivity of the RGD-p21Ras-scFv recombinant antibody with K-p21RAS, H-p21RAS, and N-p21RAS, we examined the immunobinding ability of the purified scFv with K-p21RAS, H-p21RAS, and N-p21RAS using ELISA, and the results showed that 1 mg/ml of RGD-p21Ras-scFv binds to K-p21Ras, H-p21Ras, and N-p21Ras proteins with an immunoreactive activity of 1:800 (Fig. 2A). In addition, we verified the binding activity of RGD-p21Ras-scFv with K-p21Ras, H-p21Ras and N–p21Ras by molecular docking, and the results showed that RGD-p21Ras-scFv binds to amino acid residues of K-p21Ras, H-p21Ras and N-p21Ras proteins mainly through hydrogen bonding and salt bridges, with the total binding free energies were − 48.81 kcal/mol, -39.27 kcal/mol and − 35.13 kcal/mol, respectively. (Fig. 2B and D).

Finally, our study further explored the stability of RGD-p21Ras-scFv binding to K-p21Ras, H-p21Ras and N-p21Ras proteins by molecular dynamics simulations. Our simulation process has a total of 5,000,000 steps with a step size of 2 fs and a total time of 100ns. Through simulations, we found that the RMSF value of RGD-p21Ras-scFv fluctuated between 0.15 and 0.75 nm, the RMSF value of K-p21Ras fluctuated between 0.15 and 0.60 nm, the RMSF value of H-p21Ras fluctuated between 0.15 and 0.85 nm, and the RMSF value of N-p21Ras fluctuated between 0.15 and 0.58 nm, indicating that RGD-p21Ras-scFv and K-p21Ras, H-p21Ras, and N-p21Ras proteins had minimal fluctuations in the root mean square displacement and average conformation per amino acid, and had good mutual binding activity (Fig. 2E and G). The RMSD values of RGD-p21Ras-scFv with K-p21Ras protein complex fluctuated between 0.2 and 0.6 nm, the RMSD values of RGD-p21Ras-scFv with H-p21Ras protein complex fluctuated between 0.15 and 0.6 nm, the RMSD values of RGD-p21Ras-scFv with N-p21Ras protein complex fluctuated between 0.15 and 0.82 nm indicating that the complex fluctuated very little throughout the kinetic simulation and remained stable within a suitable range. The RMSD values of RGD-p21Ras-scFv complex with N-p21Ras protein fluctuated between 0.15 and 0.82 nm indicating that the complexes fluctuated very little throughout the kinetic simulation, were in equilibrium, and remained stable within a suitable range (Fig. 2H J). Also, we analyzed the Rg (radius of gyration) of RGD-p21Ras-scFv in complex with K-p21Ras, H-p21Ras, and N-p21Ras proteins by molecular dynamics simulations, and we found that the Rg values of RGD-p21Ras-scFv in complex with K-p21Ras protein fluctuated between 2. 15 and 2.25 nm, the RGD- p21Ras-scFv fluctuated between 2.10 and 2.25 nm for the Rg value of the RGD-p21Ras-scFv with the H-p21Ras protein complex, and 2.15–2.35 nm for the RGD-p21Ras-scFv with the N-p21Ras protein complex, suggesting that the Rg values of RGD-p21Ras-scFv with the K-p21Ras, H- p21Ras and N-p21Ras proteins formed a stable complex (Fig. 2K and M). During the simulations, RGD-p21Ras-scFv formed an average of 6–10 hydrogen bonds upon binding to K-p21Ras, H-p21Ras, and N-p21Ras, which represents a strong interaction of the complex (Fig. 2N and P).

### RGD-p21Ras-scFv significantly inhibits the growth of multiple ras-derived Tumor cell lines

**Fig. 3:**
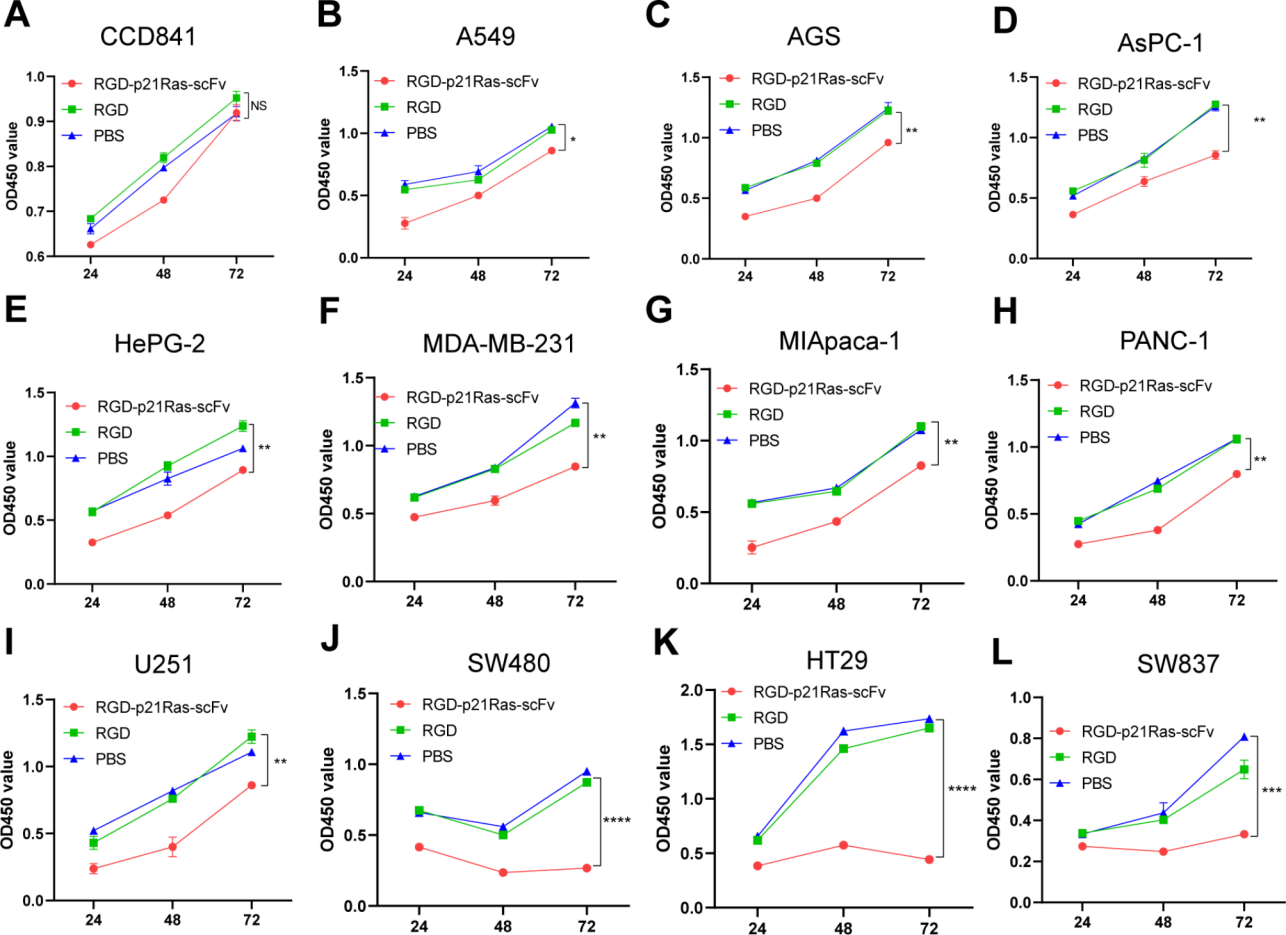
RGD-p21Ras-scFv significantly inhibits the growth of Ras-derived tumor cell lines. Eleven Ras-derived-associated tumor cell lines and one normal epithelial cell lines were co-cultured with RGD-p21Ras-scFv, PBS, and RGD peptide in 96-well plates for 24, 48, and 72 h, and then cellular activity was assayed using the CCK-8 time. rgd-p21Ras-scFv inhibited the growth of the incorporated Ras-derived tumor cells but had no normal colon epithelial cell effects. **(A)** CCD841; **(B)** A549, **(C)** AGS, **(D)** AsPC-1, **(E)** HePG-2, **(F)** MDB-MA-321, **(G)** MIApaca-2, **(H)** PANC- 1, **(I)** U251, **(J)** SW480, **(K)** HT29 and (L) SW480 Data are expressed as the mean of three independent experiments expressed, Mean ± SD. *p < 0.05; **p < 0.01, ***p < 0.001, ****p < 0.0001

To determine the antitumor activity of RGD-p21Ras-scFv in vitro, we used 30 µM PBS, RGD peptide, and 0.32 µM RGD-p21Ras-scFv with Ras-derived tumor cell lines (A549, AGS, AsPC-1, HePG-2, MDB-MA-321, MIApaca-2, PANC- 1, U 251, SW480, HT29 and SW480) and a normal colon epithelial cell line (CCD841) were co-cultured for a period of time, and cell proliferation was analyzed by CCK-8 assay. The results showed that neither PBS, RGD nor RGD-p21Ras-scFv inhibited the growth of the normal colon epithelial cell line CCD841 (Fig. 3A); however, RGD-p21Ras-scFv significantly inhibited the growth of all the Ras-associated tumor cell lines we included (Fig. 3B L). Importantly, it is evident from our results that RGD-p21Ras-scFv inhibited colorectal cancer cell lines most significantly and showed a more statistically significant difference (Fig. 3J L). Therefore in the following study we used the tumor cell lines expressing the most common KRAS origin in colorectal cancer as our in-depth study, and also included more KRAS mutated types of colorectal cancer cell lines.

### RGD-p21Ras-scFv enters KRAS wild-type and mutant Colorectal cancer cell to bind to p21Ras

**Fig. 4:**
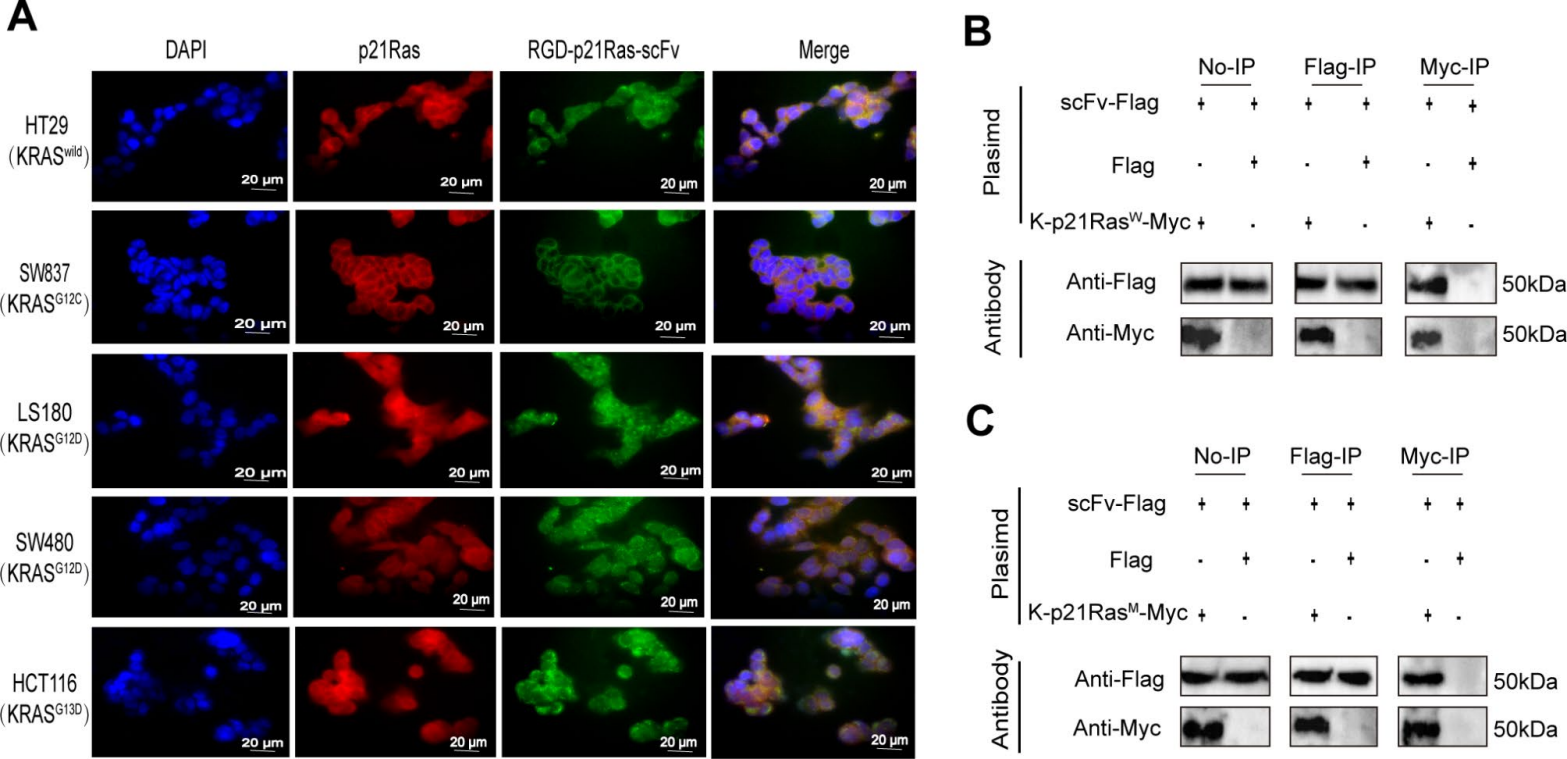
RGD-p21Ras-scFv enters tumor cells to bind directly to p21Ras. **(A)** Immunofluorescence assay showed that RGD-p21Ras-scFv(green) entered tumor cells and co-localized with p21Ras(red) in colorectal cancer cell lines HT29, SW837, LS180, SW480, HCT116. Nuclei were stained with DAPI (blue). **(B, C)** Pull-down assay revealed that RGD-p21Ras-scFv could directly bind with wild-type K-p21Ras and mutant K-p21Ras. Plasmids of RGD-p21Ras-scFv with Flag (scFv-Flag), blank with Flag (Flag), wild-type KRAS with MYC (K-p21RasW-Myc), and mutant KRAS with MYC (K-p21RasM-Myc) were transfected into 293T cells. No-IP: whole-cell lysate; Flag-IP: eluate by anti-Flag beads, Myc-IP: eluate by anti-Myc beads. No-IP, Flag-IP, and Myc-IP by WB detection

To determine the localization and distribution of RGD-p21Ras-scFv in KRAS wild-type and (KRAS^G12C^, KRAS^G12D^, KRAS^G12V^, KRAS^G13D^) mutant colorectal cancer cell lines, we performed analysis by immunofluorescence co-localization and showed that RGD-p21Ras-scFv could enter into the cancer cell membrane and co-localize with p21Ras co-localization (Fig. 4A). Meanwhile, we further validated that RGD-p21Ras-scFv interacts with KRAS wild-type and KRAS^G12C^ mutant in cells using a pull-down assay. By co-transfecting RGD-p21Ras-scFv with FLAG tag and blank plasmid with FLAG tag, RGD-p21Ras-scFv with FLAG tag and KRAS wild-type with MYC tag, and RGD-p21Ras-scFv with FLAG tag and KRAS^G12C^ mutant with MYC tag into 293T cells, respectively, after successful transfection, cell proteins were collected and FLAG- and MYC-tagged magnetic beads were added for IP experiments. The results showed that RGD-p21Ras-scFv could directly have a mutual effect with wild-type K-p21Ras and mutant K-p21Ras^G12C^. Finally, when we used FLAG and MYC antibodies for detection, we found that in IP-treated proteins we were able to detect RGD-p21Ras-scFv using FLAG antibody to detect RGD-p21Ras-scFv as well as MYC antibody; and at the same time, we were able to detect p21Ras-scFv using MYC antibody as well as RGD-p21Ras-scFv using FLAG antibody (Fig. 4B, 4 C).

### RGD-p21Ras-scFv reduced p21Ras-GTP expression and inhibited MEK-ERK/PI3K-AKT phosphorylation

**Fig. 5:**
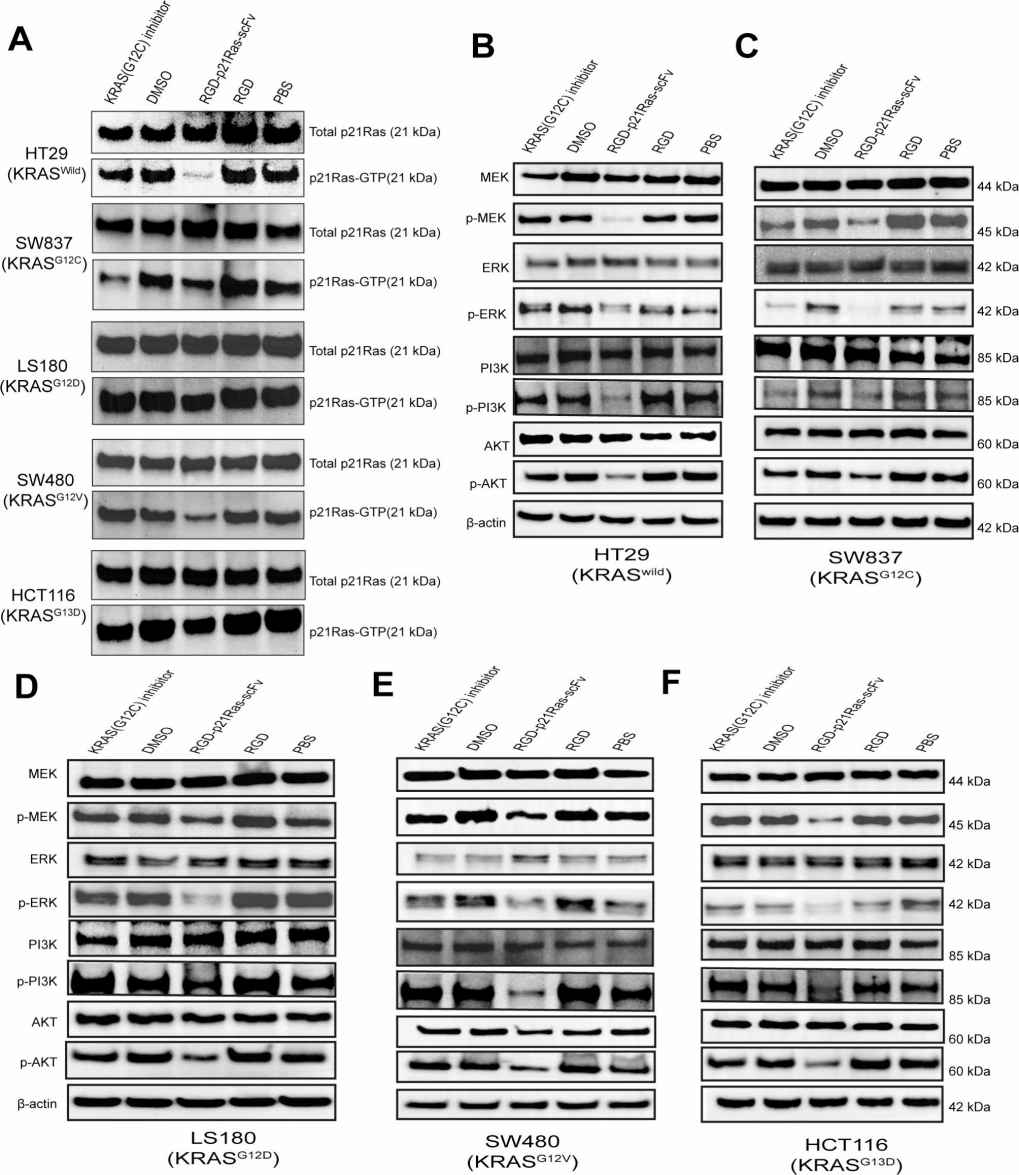
The binding between RGD-p21Ras-scFv and p21Ras protein could reduce p21Ras-GTP (active Ras) and inhibit phosphorylation of the downstream pathway MEK-ERK/PI3K-AKT in colorectal cancer. **(A)** RGD-p21Ras-scFv reduces p21Ras-GTP by western-blot analysis. The colorectal cancer cell lines were cocultured with RGD-p21Ras-scFv, KRAS(G12C) inhibitor, PBS, RGD or DMSO for 48 h. The proteins p21Ras-GTP and total p21Ras were enriched in the cell lysates by an RAS pull-down kit. **(B-F)** The RGD-p21Ras-scFv inhibits the phosphorylation of the MEK-ERK/PI3K-AKT signaling pathway. Colorectal cancer cell lines were cocultured with RGD-p21Ras-scFv, KRAS(G12C) inhibitor, PBS, RGD or DMSO for 48 h. Cells were lysed, and proteins were extracted for western blot analysis Ras changes in downstream signaling pathways. β-actin was adopted as the control

To elucidate the potential mechanism by which RGD-p21Ras-scFv inhibits the activity of colorectal cancer cell lines after stable binding to p21Ras. This study performed Ras pull-down assays using the KRAS wild-type colorectal cancer cell line HT29 and KRAS mutant colorectal cancer cell lines SW837^G12C^, LS180^G12D^, SW480^G12V^, and HCT116^G13D^. The results showed that RGD-p21Ras-scFv reduced p21Ras-GTP expression in all of the above colorectal cancer cell lines, while KRAS (G12C) inhibitor only reduced p21Ras-GTP expression in KRAS^G12C^ mutant colorectal cancer cells (Fig. 5A); after analyzing relative protein expression for quantification, our results proved to be statistically significance (Figure Supplementary 3 A).

The MEK-ERK and PI3K-AKT signaling paths downstream of Ras are the classical pathways that promote tumor progression. To further explore the effect of RGD-p21Ras-scFv on downstream signaling pathways after inhibition of Ras activity, we analyzed the changes of MEK-ERK and PI3K-AKT signaling molecules in the above KRAS wild and KRAS mutant colorectal cancer cell lines by WB. The results showed that RGD-p21Ras-scFv significantly reduced the phosphorylation of MEK-ERK/PI3K-AKT and inhibited the activation of MEK-ERK/PI3K-AKT signaling pathway in KRAS wild-type and mutant colorectal cancer cell lines, and KRAS (G12C) inhibitors only inhibited KRAS^G12C^ mutant colorectal cancer cell lines (Fig. 5B F, Supplementary Fig. 2B-F).

### RGD-p21Ras-scFv inhibits Tumor growth in nude mice

**Fig. 6:**
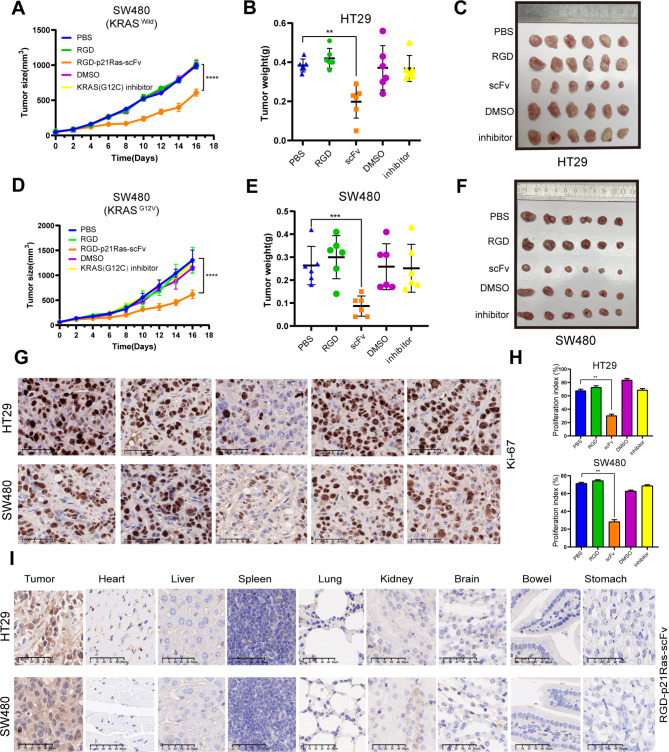
RGD-p21Ras-scFv inhibited the growth of xenografts in nude mice. **(A, D)** All the nude mice were weighed and divided into the following five groups: PBS, RGD, RGD-p21Ras-scFv, DMSO and K-Ras(G12C) inhibitor group. The tumor growth curves were drawn according to the tumor sizes. The tumor volume growth was inhibited by the RGD-p21Ras-scFv. **(B, E)** RGD-p21Ras-scFv treatment group had the lowest tumor weight compared to the other groups. **(C, F)** Tumor tissues from each group were dissected and examined by general observation. **(G, H)** IHC showed there were less Ki67-positive cells in the RGD-p21Ras-scFv treatment group. Ki67 index (PI) was calculated and scored from the number of Ki67-positive cells to the total number of tumor cells, Mean ± SD. *P < 0.05, **P < 0.01. **(I)** After 24 days of treatment, RGD-p21Ras-scFv was only presented in xenograft tumor tissues rather than in any other major organs by IHC analysis

To decide the antitumor activity of RGD-p21Ras-scFv in nude mice, we selected the KRAS wild-type colorectal cancer cell line HT29 and the KRAS^G12V^ mutant colorectal cancer cell line SW480 with low IC50 values to establish nude mouse xenograft model and administered the drug for treatment. Reduced growth rate of xenografts volume in RGD-p21Ras-scFv treated nude mice was found by plotting the tumor volume growth curve (Fig. 6A and D). At the end of the administration, individual organs and xenografts were collected and the xenografts were weighed and found that the weight of xenografts in RGD-p21Ras-scFv treated nude mice was significantly lower than other treatment groups (Fig. 6B and E), while we observed the smallest xenografts in the RGD-p21Ras-scFv treated group (Fig. 6C, 6 F).

Finally, we verified whether RGD-p21Ras-scFv could target nude mice xenografts cells and inhibit their cell proliferation, and we collected xenografts from each treatment group of nude mice for Ki67 staining and analyzed other organs of nude mice for HE staining. Ki-67 results showed that RGD-p21Ras-scFv effectively inhibited the proliferation index of xenografts established in human colorectal cancer cell lines in nude mice (Fig. 6G, 6 H). HE staining showed that RGD-p21Ras-scFv treatment did not cause pathological damage to the major organs of nude mice (Supplementary Fig. 3A, 3B). Moreover, we analyzed the distribution of RGD-p21Ras-scFv in the organs of nude mice, and immunohistochemical results showed that RGD-p21Ras-scFv was present only in xenograft (Fig. 6I), while the same results were obtained by WB assay (Supplementary Fig. 4).

## Discussion

In previous studies, we constructed RGD-p21Ras scFv recombinant antibody and expressed them in small amounts in E. coli in the laboratory. It was found that the RGD-p21Ras scFv could enter the tumor cells that has integrin αvβ3 expression on the surface and bind to intracellular p21Ras to inhibit the proliferation of tumor cells. However, the key to clinical translational application of p21Rras recombinant antibody is to scale expression and maintain their biological effects of penetrating into tumor cells and binding to intracellular p21Ras.

In this study, we optimized the conditions for the expression of recombinant proteins to solve the problem of obtaining large amounts of proteins. It can conduct in vivo experiments and prepare for future preclinical studies to scale up protein production. When RGD peptide conjugated with proteins, they may result in low production. For example, Curnis et al [30]. used RGD to modify TNF, resulting in getting 2 mg of RGD-MTNF from 1 L of *E. coli.* In our study, through the selection of different plasmids and bacteria, we screened the best expression plasmid and bacteria combination. ZYM-5052 culture medium can further improve the expression level of target protein, and the result was demonstrated that the self-induced expression of ZYM-5052 is much higher than that of the traditional IPTG-induced expression. Through the fermentation culture of 60 L in a 100 L bioreactor, we can finally get 31.064 g/L wet-weight bacteria. Compared with 10.6 g/L under laboratory conditions, it is significantly improved. Compared with the 30 g/L wet bacteriophage obtained from the pilot-scale preparation process established by the SA-hGM-CSF bifunctional fusion protein, our recombinant antibody was able to meet the requirements of the pilot-scale preparation [31].

In *E. coli.* expression systems, purification and renaturation are essential to obtain biologically active proteins. Moreover, the greatest loss of protein occurs usually in these two steps. Unlike other studies [32], a two step purification method was used in our study. Firstly, we used affinity chromatography under denaturation conditions, and by gradient refolding to remove urea and imidazole. Then, DEAE ion exchange chromatography resin was used to further purify the protein after refolding, which was beneficial to improve the purity. After refolding and DEAE purification, the protein with low binding affinity was flow-through, and about 60% of the protein was obtained. Finally, Our protein purity is higher than 85%. The purity was in the same range as other antibody purification obtained from other reports [33, 34], which proved that our purification was effective.

It is certain that our preparation of RGD-p21Ras-scFv has strong immunoreactivity with p21Ras (K-p21Ras, N-p21Ras, H-p21Ras) and can bind to them to exert anti-tumor effects. We visualized that scale expression of RGD-p21Ras-scFv entered KRAS wild and KRAS mutant colorectal cancer cell lines and co-localized with p21Ras protein by immunofluorescence. Second, the analysis of performing pull-down tests also directly illustrates that our RGD-p21Ras-scFv interacts with wild K-p21Ras and mutant K-p21Ras proteins. Further, we also used molecular docking and molecular dynamics simulations to demonstrate that RGD-p21Ras-scFv has strong binding ability with wild K-p21Ras and mutant K-p21Ras proteins, and the bound protein complexes remain stable for a longer period of time. Here, we can clarify that scale expression of RGD-p21Ras-scFv enters tumor cells and binds to p21Ras by multiple forces including hydrogen bonding, and the bound complex remains stable for a long time.

As it is known that p21Ras switches between binding to GDP (inactive) and GTP (active), we have clarified that scale expression of RGD-p21Ras-scFv can bind to p21Ras protein with a very significant interaction, it was observed that RGD-p21Ras-scFv can reduce the expression of active p21Ras (p21Ras-GTP) protein after binding to p21Ras protein by Ras pull down assay. Also, considering that active p21Ras activates MEK phosphorylation, which activates downstream ERK phosphorylation [35], activation of PI3K/AKT phosphorylation also promotes RAS-dependent tumor growth and exerts a complementary effect on the MEK/ERK signaling cascade [36]. Thus, this study also demonstrated that RGD-p21Ras-scFv reduced activity of p21Ras after downregulating the phosphorylation of MEK-ERK/PI3K-AKT signaling pathway downstream of Ras. Based on these results, we elucidated for the first time the specific mechanism by which RGD-p21Ras-scFv exerts its antitumor activity: RGD-p21Ras-scFv enters tumor cells and binds to p21Ras, reduces the expression of active p21Ras (p21Ras-GTP) protein, and further inhibits the phosphorylation of MEK-ERK/PI3K-AKT signaling pathway.

We found that scale expression of RGD-p21Ras-scFv have anti-tumor activity in vitro, since many drugs were developed with good in vitro effects but poor in vivo effects and poor safety [37], we selected the KRAS wild-type colorectal cancer cell line HT29 and the KRAS^G12V^ mutant colorectal cancer cell line SW480 with good in vitro effects to establish a nude mouse xenograft tumor model and treated with RGD-p21Ras-scFv administration. We found that scale expression of RGD-p21Ras-scFv greatly stopped the development of xenograft tumors while ensuring a certain safety profile, specifically targeting xenograft tumors and reducing the proliferation of tumor cells.

In conclusion, we screened the combination of recombinant plasmids and expression bacteria as well as optimized the induction expression conditions and successfully established the pilot-scale prokaryotic expression of RGD-p21Ras-scFv recombinant antibody. Furthermore, we elucidated for the first time that scale expression of RGD-p21Ras-scFv enters KRAS wild and mutant colorectal cancer cell lines to stably bind to p21Ras and reduce the expression of active p21Ras protein, and consequently inhibits phosphorylation of the MEK-ERK/PI3K-AKT signaling pathway downstream of Ras, providing safety while effectively inhibiting RAS-dependent tumor growth. Our outcomes offer a theoretical basis for the clinical translation and usage of RGD-p21Ras-scFv for the treatment of Ras-derived tumors.

### Electronic supplementary material

Below is the link to the electronic supplementary material.

Supplementary Material 1

## Data Availability

The protein structure data analyzed in this study can be found in the RCSB Protein Data Bank at https://www.rcsb.org/ PDB files are (K-p21Ras, ID: 4LDJ; H-p21Ras, ID: 6E6P; N-p21Ras, ID: 3CON). Also, information supporting the results of this study can be found in the article or in the supplementary material and is available from the corresponding author on request.

## References

[1] Parada LF, Tabin CJ, Shih C, Weinberg RA (1982). Human EJ bladder carcinoma oncogene is homologue of Harvey sarcoma virus ras gene. Nature.

[2] Emel’ianova MA, Amosenko FA, Chudinov AV, Surzhikov SA, Kazubskaia TP, Liubchenko LN, Nasedkina TV (2011). [Detection of KRAS mutations in Tumor cells using biochips]. Mol Biol (Mosk).

[3] Zinatizadeh MR, Momeni SA, Zarandi PK, Chalbatani GM, Dana H, Mirzaei HR, Akbari ME, Miri SR (2019). The role and function of ras-association domain family in Cancer: a review. Genes Dis.

[4] Drosten M, Barbacid M (2020). Targeting the MAPK pathway in KRAS-Driven tumors. Cancer Cell.

[5] Bai S, Feng Q, Pan XY, Zou H, Chen HB, Wang P, Zhou XL, Hong YL, Song SL, Yang JL (2017). Overexpression of wild-type p21Ras plays a prominent role in Colorectal cancer. Int J Mol Med.

[6] Hofmann MH, Gmachl M, Ramharter J, Savarese F, Gerlach D, Marszalek JR, Sanderson MP, Kessler D, Trapani F, Arnhof H (2021). BI-3406, a potent and selective SOS1-KRAS Interaction inhibitor, is effective in KRAS-Driven cancers through combined MEK Inhibition. Cancer Discov.

[7] Thein KZ, Biter AB, Hong DS (2021). Therapeutics targeting mutant KRAS. Annu Rev Med.

[8] Canon J, Rex K, Saiki AY, Mohr C, Cooke K, Bagal D, Gaida K, Holt T, Knutson CG, Koppada N (2019). The clinical KRAS(G12C) inhibitor AMG 510 drives anti-tumour immunity. Nature.

[9] Dueling, KRAS(G12C) (2020). Inhibitors achieve responses. Cancer Discov.

[10] Furth ME, Davis LJ, Fleurdelys B, Scolnick EM (1982). Monoclonal antibodies to the p21 products of the transforming gene of Harvey murine sarcoma virus and of the cellular ras gene family. J Virol.

[11] Yoshida K, Hamatani K, Koide H, Ikeda H, Nakamura N, Akiyama M, Tsuchiyama H, Nakayama E, Shiku H (1988). Preparation of anti-ras Mr 21,000 protein monoclonal antibodies and immunohistochemical analyses on expression of ras genes in human stomach and thyroid cancers. Cancer Res.

[12] Yang JL, Liu DX, Zhen SJ, Zhou YG, Zhang DJ, Yang LY, Chen HB, Feng Q (2016). A novel anti-p21Ras scFv antibody reacting specifically with human tumour cell lines and primary tumour tissues. BMC Cancer.

[13] Yang JL, Pan XY, Zhao WX, Hu QC, Ding F, Feng Q, Li GY, Luo Y (2016). The antitumor efficacy of a novel adenovirus-mediated anti-p21Ras single chain fragment variable antibody on human cancers in vitro and in vivo. Int J Oncol.

[14] Pan XY, Liu XJ, Li J, Zhen SJ, Liu DX, Feng Q, Zhao WX, Luo Y, Zhang YL, Li HW (2017). The antitumor efficacy of anti-p21Ras scFv mediated by the dual-promoter-regulated recombinant adenovirus KGHV300. Gene Ther.

[15] Liu FR, Bai S, Feng Q, Pan XY, Song SL, Fang H, Cui J, Yang JL (2018). Anti-colorectal cancer effects of anti-p21Ras scFv delivered by the recombinant adenovirus KGHV500 and cytokine-induced killer cells. BMC Cancer.

[16] Wang M, Hong Y, Feng Q, Pan X, Song S, Cui J, Lei J, Fang H, Yang J (2018). Recombinant adenovirus KGHV500 and CIK cells Codeliver Anti-p21-Ras scFv for the treatment of gastric Cancer with Wild-Type Ras Overexpression. Mol Ther Oncolytics.

[17] Lin XR, Zhou XL, Feng Q, Pan XY, Song SL, Fang H, Lei J, Yang JL (2019). CIK cell-based delivery of recombinant adenovirus KGHV500 carrying the anti-p21Ras scFv gene enhances the anti-tumor effect and safety in Lung cancer. J Cancer Res Clin Oncol.

[18] Dai F, Zhang PB, Feng Q, Pan XY, Song SL, Cui J, Yang JL (2021). Cytokine-induced killer cells carrying recombinant oncolytic adenovirus expressing p21Ras scFv inhibited Liver cancer. J Cancer.

[19] Qian J, Yang M, Feng Q, Pan XY, Yang LL, Yang JL (2021). Inhibition of glioma by adenovirus KGHV500 encoding anti-p21Ras scFv and carried by cytokine-induced killer cells. Exp Biol Med (Maywood).

[20] Huang CC, Liu FR, Feng Q, Pan XY, Song SL, Yang JL (2021). RGD4C peptide mediates anti-p21Ras scFv entry into Tumor cells and produces an inhibitory effect on the human colon Cancer cell line SW480. BMC Cancer.

[21] Yu T, Shi Y, Pan X, Feng Q, Wang P, Song S, Yang L, Yang J (2022). BR2 cell penetrating peptide effectively delivers anti-p21Ras scFv to Tumor cells with ganglioside expression for therapy of ras-driven Tumor. PLoS ONE.

[22] Du Y, Lin X, Feng Q, Pan X, Song S, Yang J (2022). Inhibition of human Lung cancer cells by anti-p21Ras scFv mediated by the activatable cell-penetrating peptide. Anticancer Drugs.

[23] Chen J, Yang H, Feng Y, Shi Q, Li Z, Tao Z, Fan J, Jin Y, Li S, Cheng J (2021). A single nucleotide mutation drastically increases the expression of tumor-homing NGR-TNFα in the E. Coli M15-pQE30 system by improving gene transcription. Appl Microbiol Biotechnol.

[24] Studier FW (2005). Protein production by auto-induction in high density shaking cultures. Protein Expr Purif.

[25] Brenke R, Hall DR, Chuang GY, Comeau SR, Bohnuud T, Beglov D, Schueler-Furman O, Vajda S, Kozakov D (2012). Application of asymmetric statistical potentials to antibody-protein docking. Bioinformatics.

[26] Abraham MJ, Murtola T, Schulz R, Páll S, Smith JC, Hess B, Lindahl E (2015). GROMACS: high performance molecular simulations through multi-level parallelism from laptops to supercomputers. SoftwareX.

[27] Van Der Spoel D, Lindahl E, Hess B, Groenhof G, Mark AE, Berendsen HJ (2005). GROMACS: fast, flexible, and free. J Comput Chem.

[28] Tian XP, Cai J, Ma SY, Fang Y, Huang HQ, Lin TY, Rao HL, Li M, Xia ZJ, Kang TB (2020). BRD2 induces drug resistance through activation of the RasGRP1/Ras/ERK signaling pathway in adult T-cell lymphoblastic Lymphoma. Cancer Commun (Lond).

[29] Keam B, Im SA, Lee KH, Han SW, Oh DY, Kim JH, Lee SH, Han W, Kim DW, Kim TY (2011). Ki-67 can be used for further classification of triple negative Breast cancer into two subtypes with different response and prognosis. Breast Cancer Res.

[30] Curnis F, Gasparri A, Sacchi A, Longhi R, Corti A (2004). Coupling Tumor necrosis factor-alpha with alphaV integrin ligands improves its antineoplastic activity. Cancer Res.

[31] Li X, Zhou S, Wang Y, Lian H, Zuo A, Zhou K, Tong L, Zhou Z, Gao J (2019). The pilot-scale preparation of the SA-hGM-CSF bi-functional fusion protein. Bioengineered.

[32] Zhang H, Wu J, Zhang Y, Fu N, Wang J, Zhao S (2010). Optimized procedure for expression and renaturation of recombinant human bone morphogenetic protein-2 at high protein concentrations. Mol Biol Rep.

[33] Zhang X, Xie J, Sun Y, Xu H, Du T, Liu Z, Chen J, Zheng Z, Liu K, Zhang J (2014). High-level expression, purification, and characterization of bifunctional ScFv-9R fusion protein. Appl Microbiol Biotechnol.

[34] Zhang S, Huang J, Zhang L, Gu J, Song Q, Cai Y, Zhong J, Zhong H, Deng Y, Zhu W (2021). Fermentation, purification, and Tumor Inhibition of a disulfide-stabilized Diabody Against Fibroblast Growth Factor-2. Front Oncol.

[35] Tsubaki M, Takeda T, Noguchi M, Jinushi M, Seki S, Morii Y, Shimomura K, Imano M, Satou T, Nishida S. Overactivation of akt contributes to MEK inhibitor primary and Acquired Resistance in Colorectal Cancer cells. Cancers (Basel) 2019, 11(12). 10.3390/cancers11121866 PMC6966459 31769426

[36] Roberts PJ, Der CJ (2007). Targeting the Raf-MEK-ERK mitogen-activated protein kinase cascade for the treatment of cancer. Oncogene.

[37] Zips D, Thames HD, Baumann M (2005). New anticancer agents: in vitro and in vivo evaluation. In Vivo.

