# Supplementary material for: RGD-p21Ras-scFv expressed prokaryotically on a pilot scale inhibits ras-driven colorectal cancer growth by blocking p21Ras-GTP
Source: BMC Cancer. 2024 Jan 12;24:71. doi: 10.1186/s12885-023-11686-5 (PMC10787443; doi:10.1186/s12885-023-11686-5)
Supplement: Supplementary file 1 — Supplementary Material 1 [file 12885_2023_11686_MOESM1_ESM.docx]

Supplementary Material

# Supplementary Figures


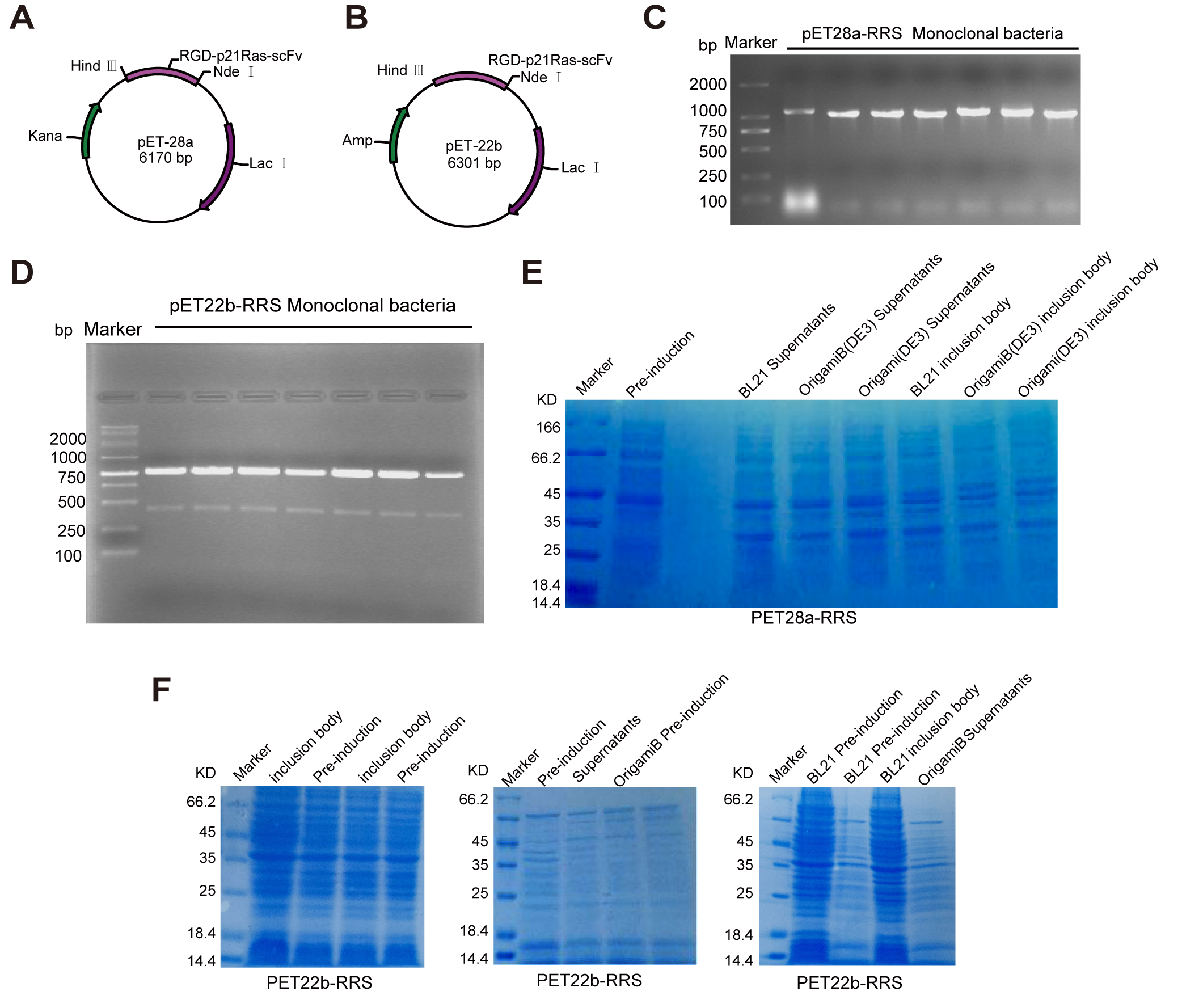


**(Supplementary Figure 1)** Screening of RGD-p21Ras-scFv recombinant antibody. The recombinant expression plasmids (A) pET28a-RRS, (B) pET22b-RRS were set up by inserting the RGD-p21Ras-scFv target gene sequence into the corresponding enzyme cleavage site of the plasmid vector; RGD-p21Ras-scFv/p Clone 007 DH5α monoclonal bacteria were picked for PCR detection, (C) RGD-p21Ras-scFv has successfully transformed the pET28a plasmid, (D) RGD-p21Ras-scFv has successfully transformed the pET22b plasmid; plasmids were transformed into different expression bacteria to induce expression of RGD-p21Ras-scFv recombinant antibody and identified by (E) Origami B(DE3)-pET28a-RRS, (F) Origami B(DE3)-pET22b-RRS by SDS-PAGE; (G) RGD-p21Ras-scFv recombinant antibody before purification.


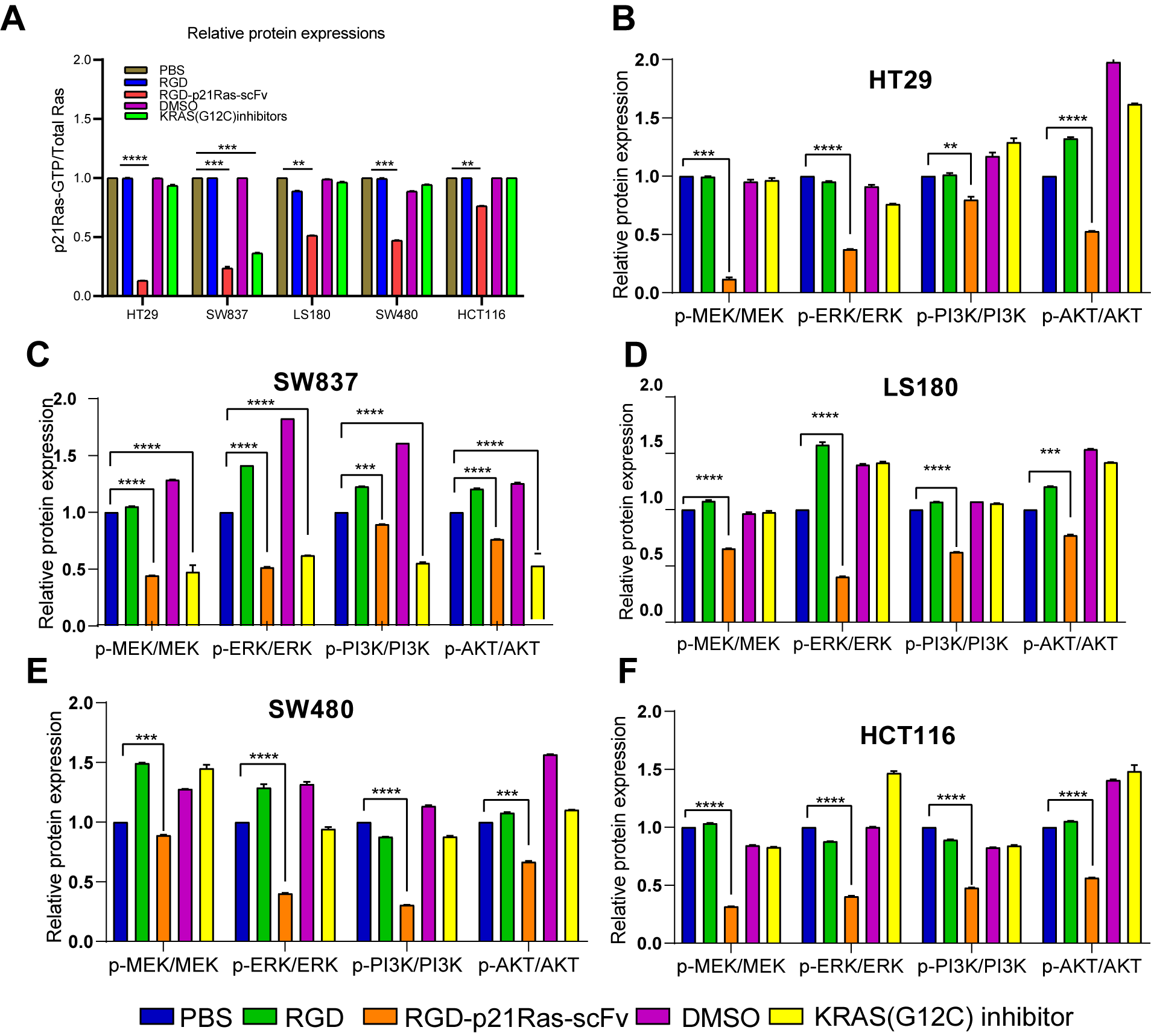


**(Supplementary Figure 2)** Their relative protein expression was analyzed and quantified by optical-density. (A)p21Ras-GTP/Total Ras; p-MEK/MEK, p-ERK/ERK, p-PI3K/PI3K, p-AKT/AKT. (B) HT29 (C) SW837;(D) LS180;(E) SW480 and (F) HCT116. Mean ±SD. *P<0.05, **P<0.01, ***P<0.001, ****P<0.0001.


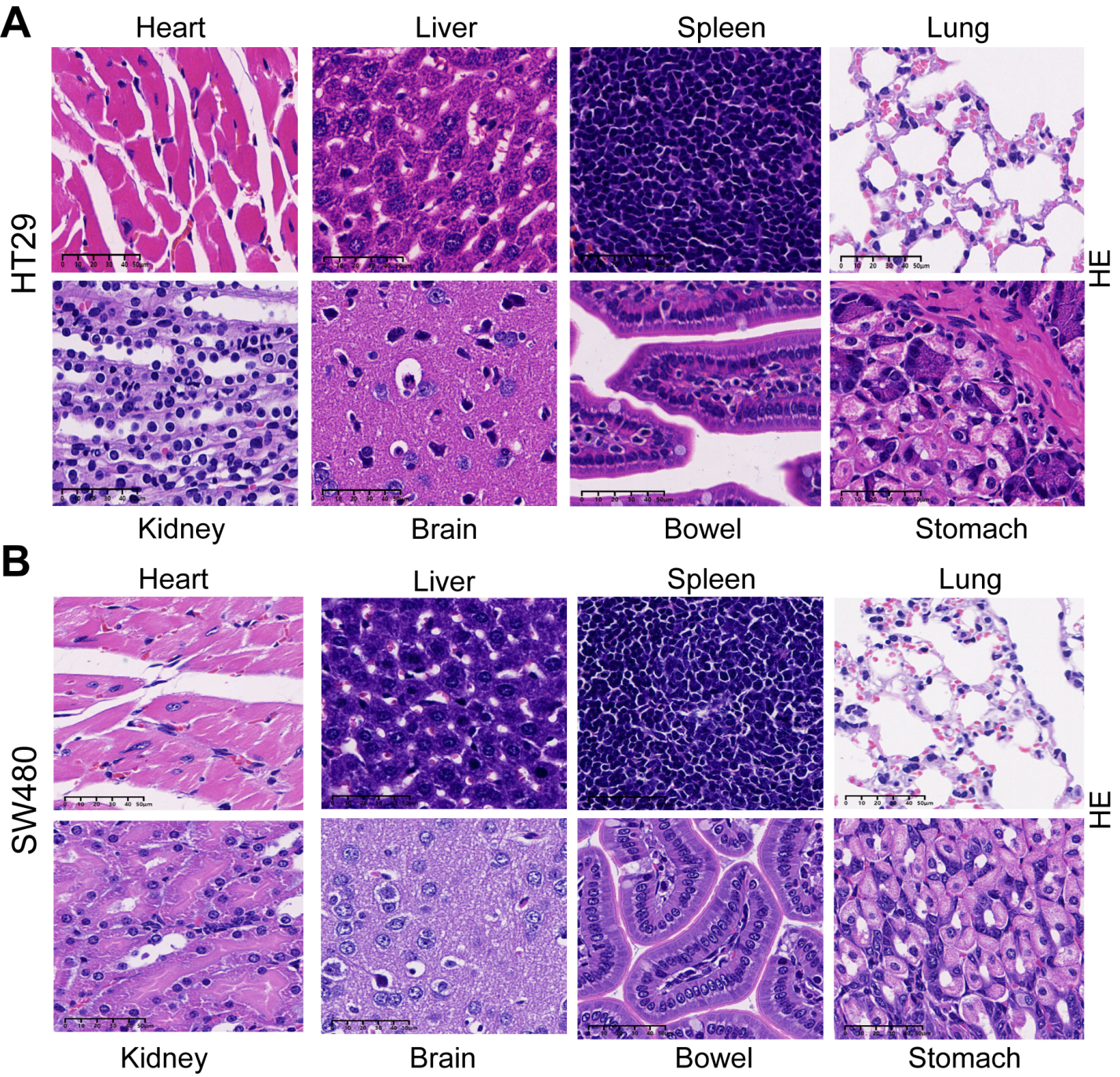


**(Supplementary Figure 3)** HE results showed no pathological damage to the major organs of nude mice by RGD-p21Ras-scFv. (A)HT29; (B)SW480.


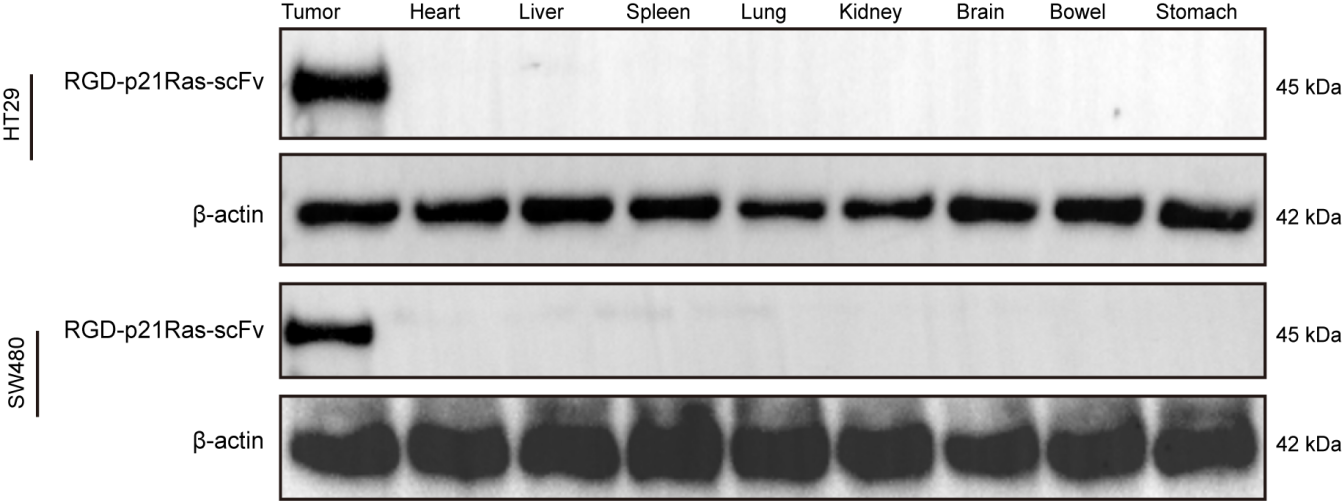


**(Supplementary Figure 4)** WB showed that RGD-p21Ras-scFv was existence only in tumor tissues and not in other tissues. β-actin was adopted as the control.


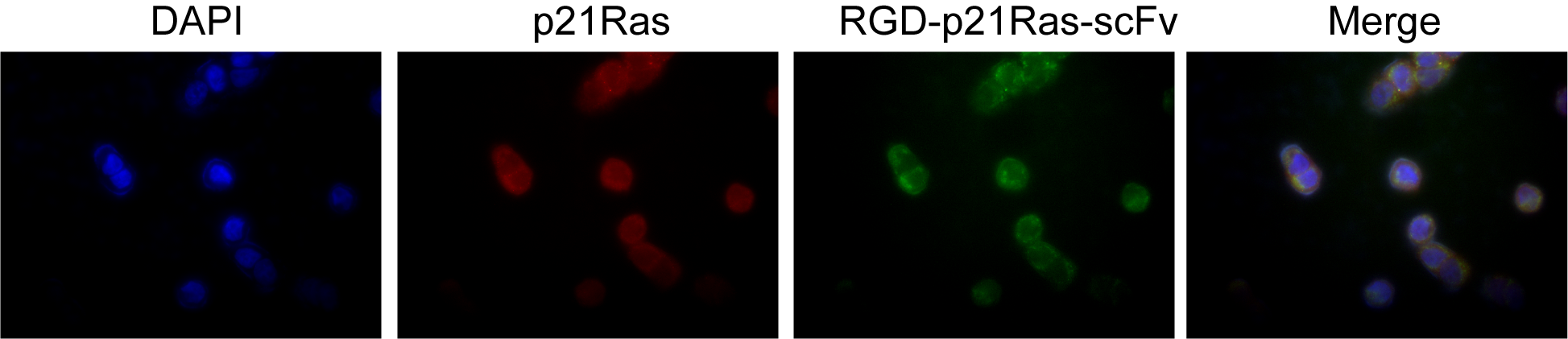


**(Supplementary Figure 5)** Immunofluorescence co-localization assay using abcam's anti-Ras antibody (ab180772) showed that RGD-p21Ras-scFv (green) enters KRAS wild-type and mutant colorectal cancer cells and co-localizes with p21Ras (red) in colorectal cancer cell line HT29. Cell nuclei were stained with DAPI (blue).

original images：

Figure.1B


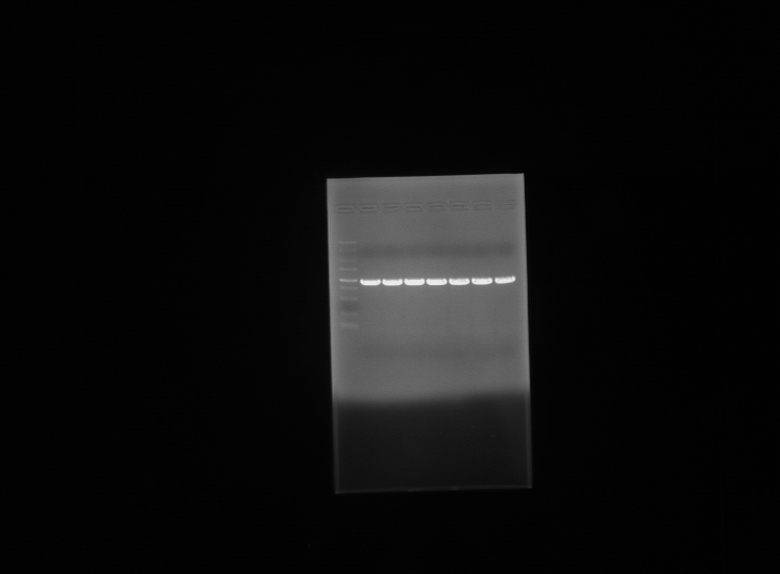


1C


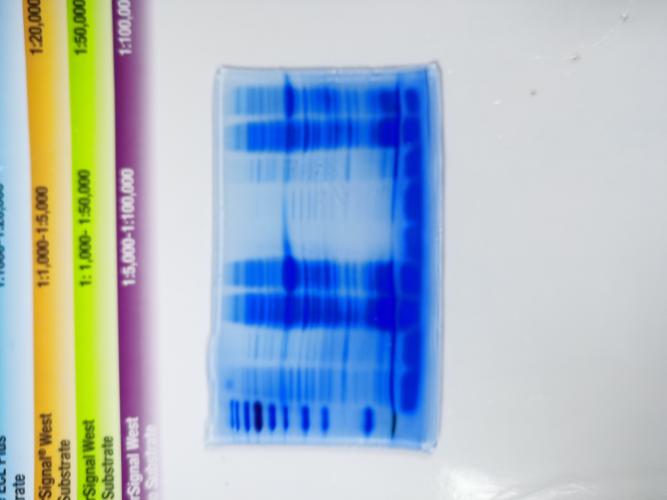


1D


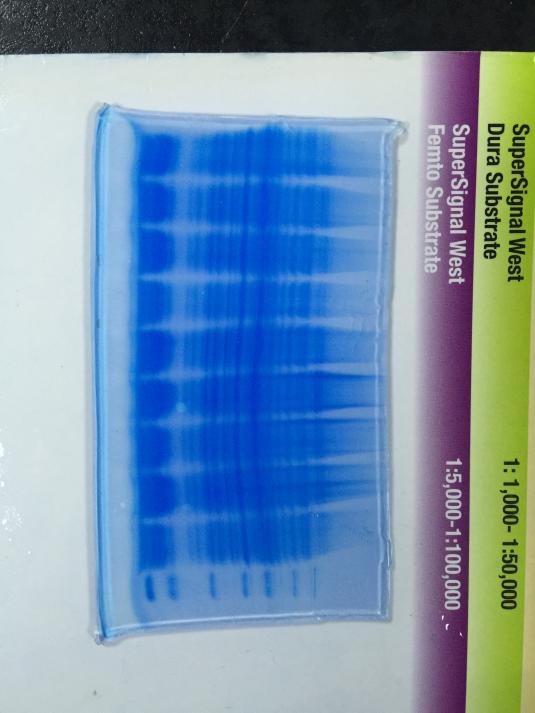


1E


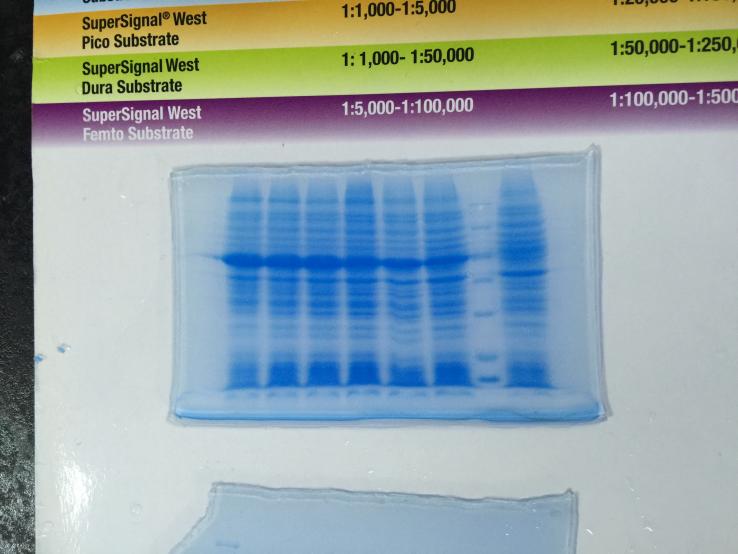


1F


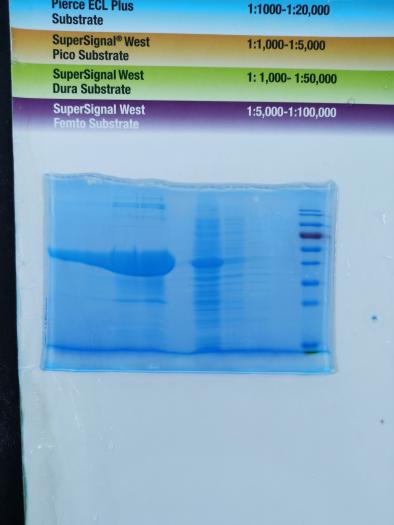


1G


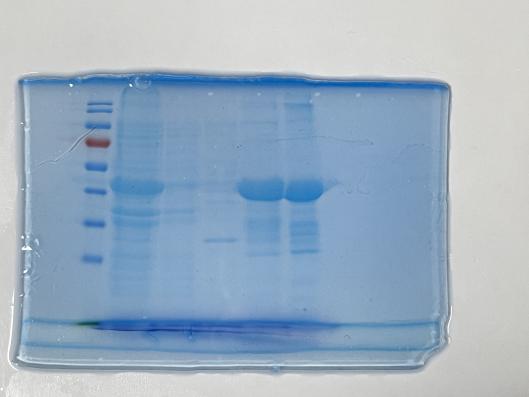


1H


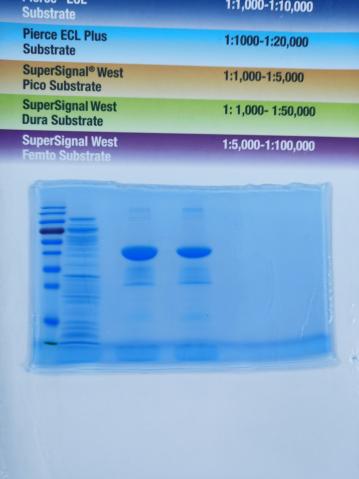


1I


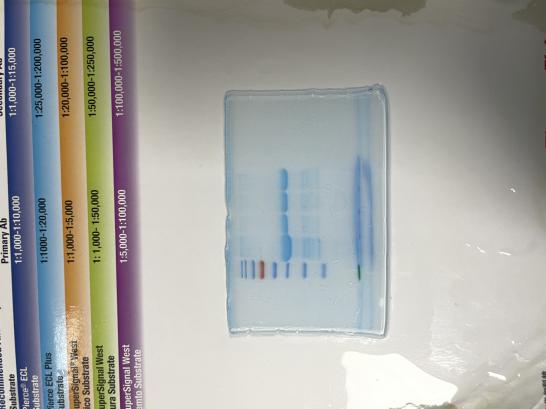


Figure.4B


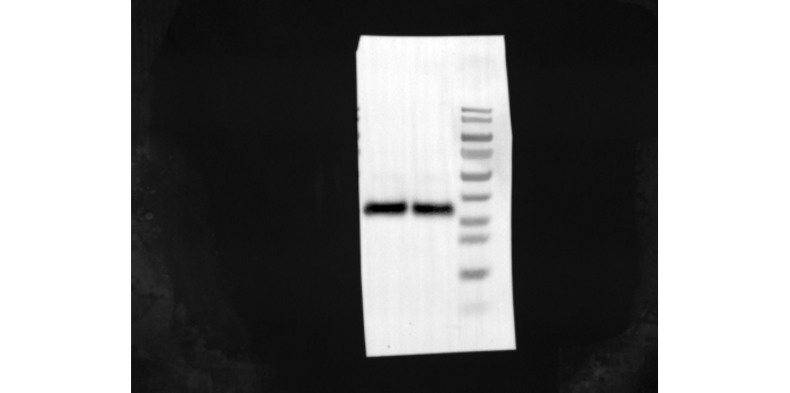


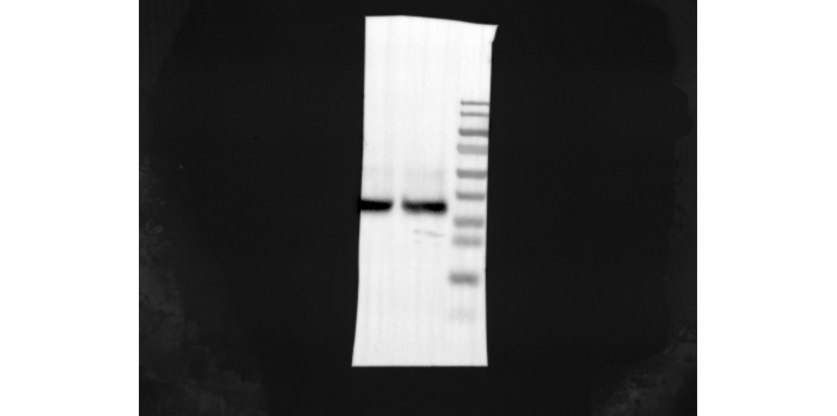


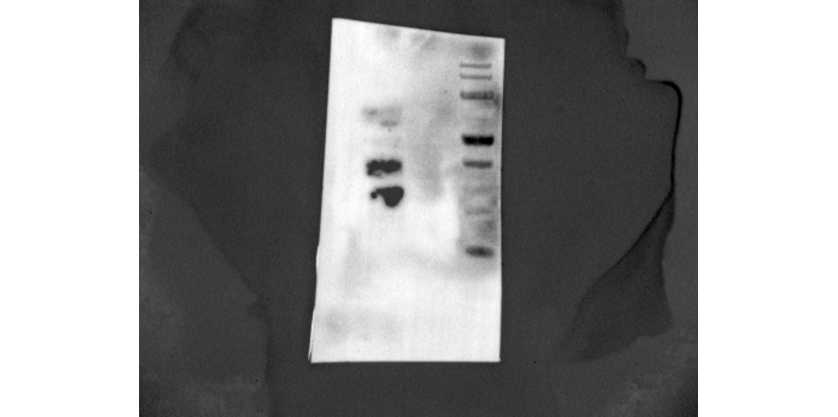


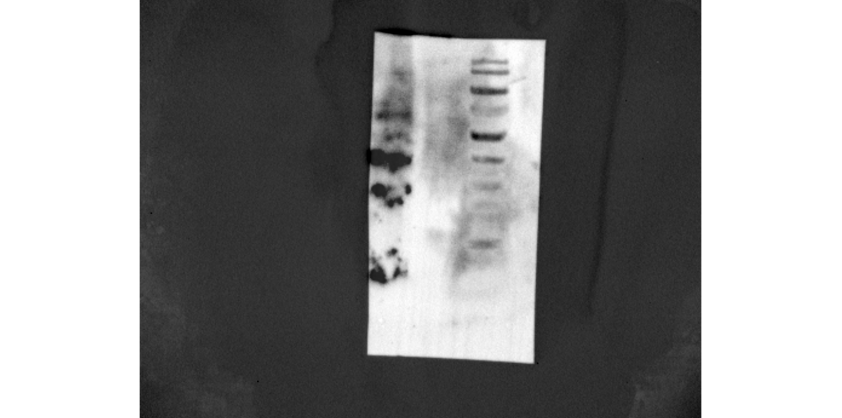


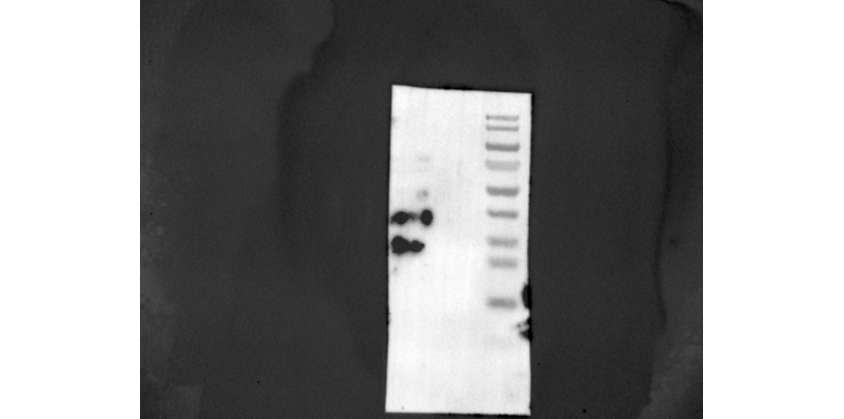


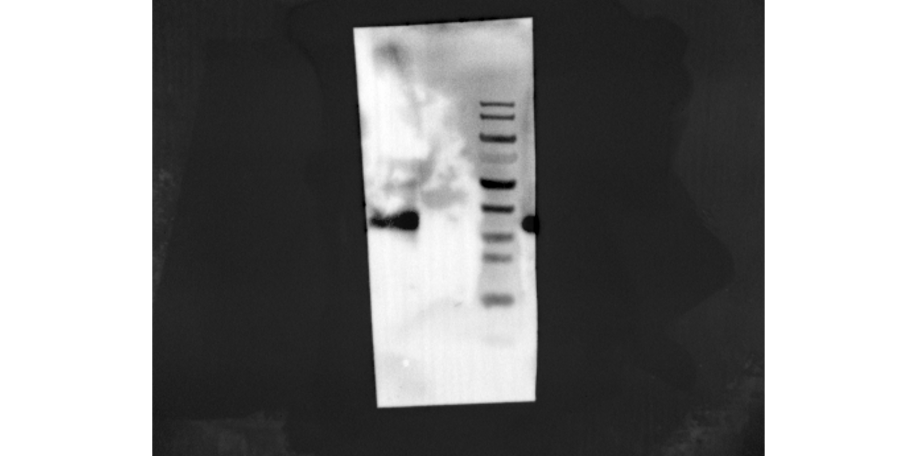


4C


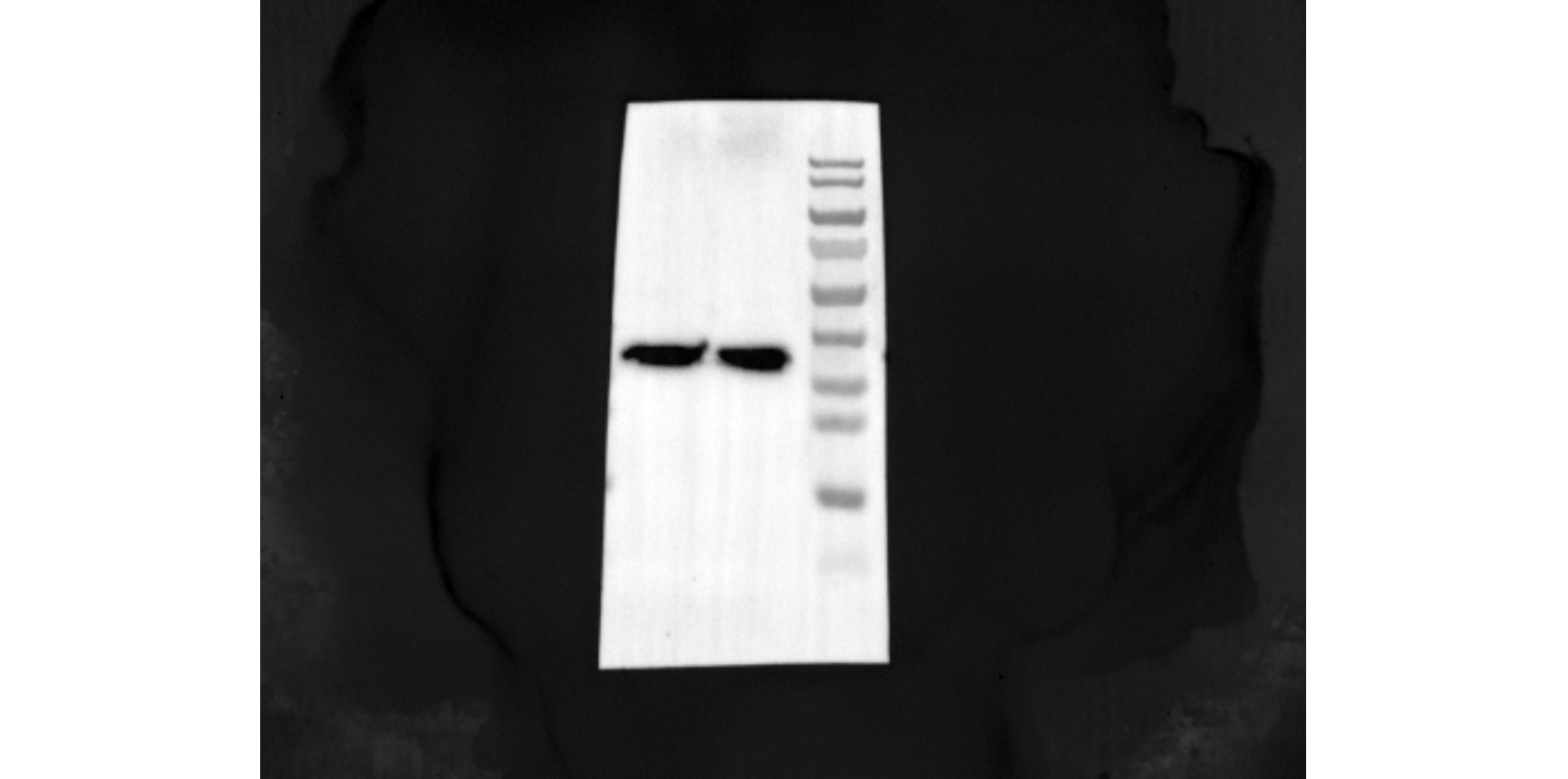


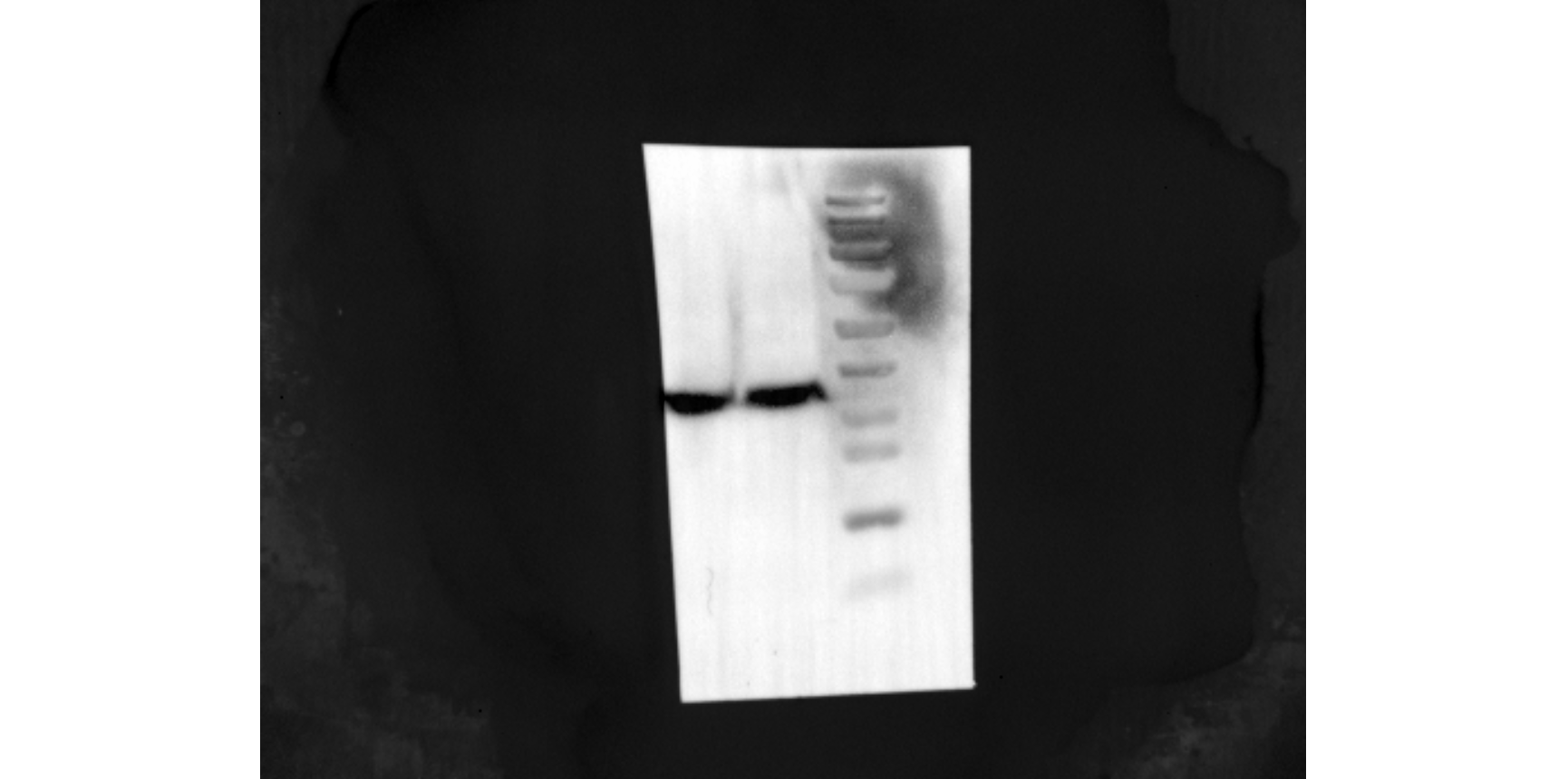


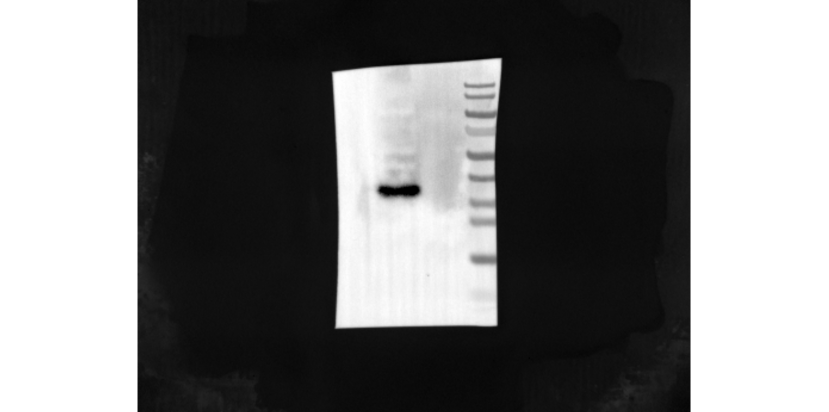


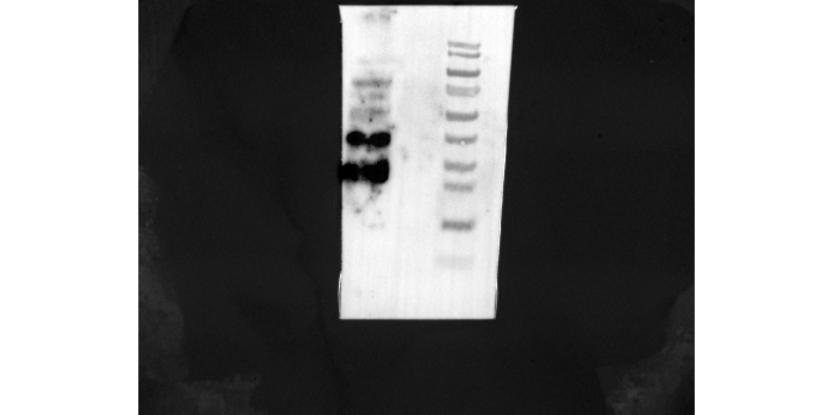


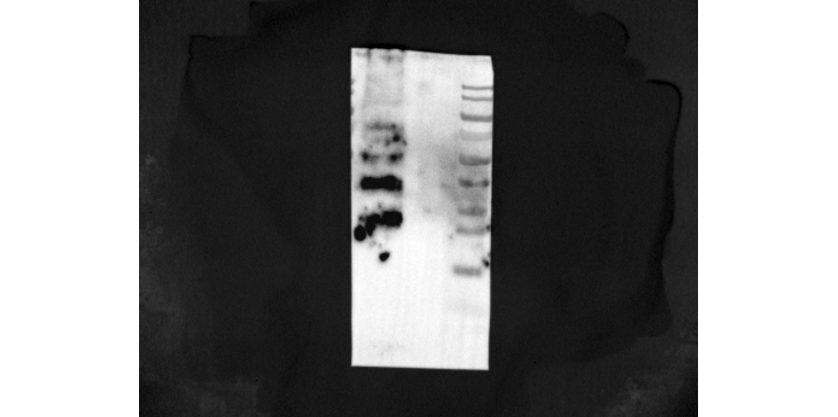


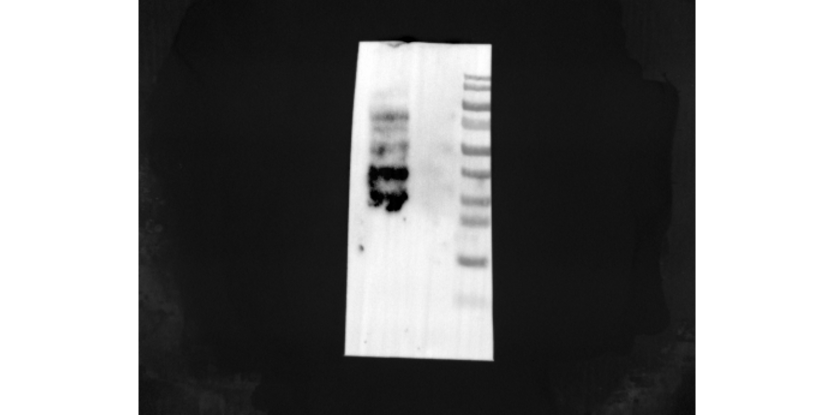


Figure.5A


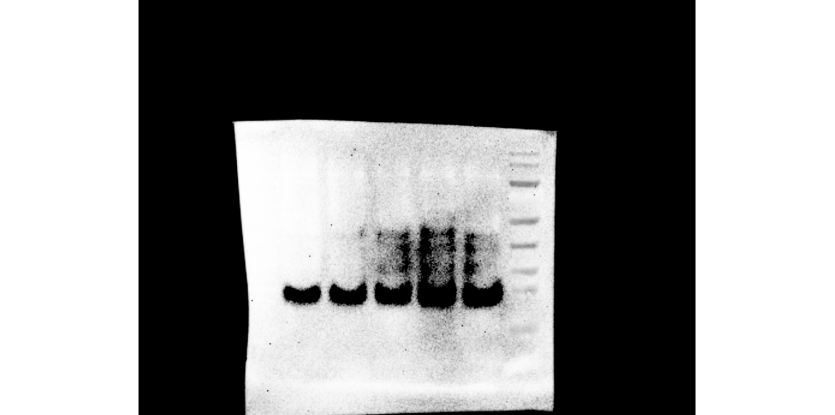
HT29 Total Ras


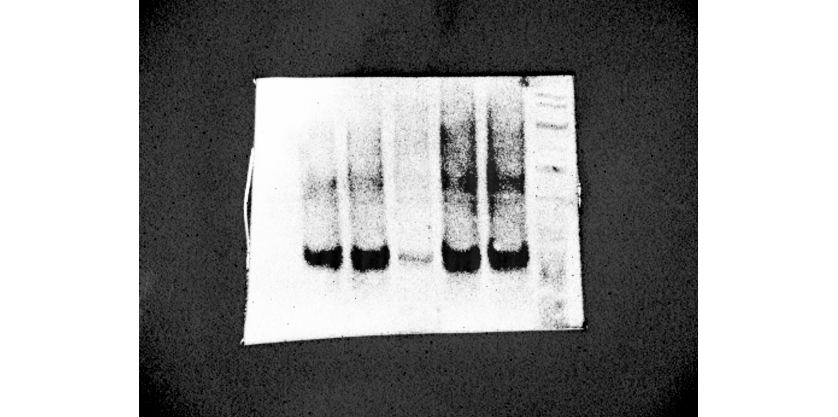
HT29 Ras-GTP


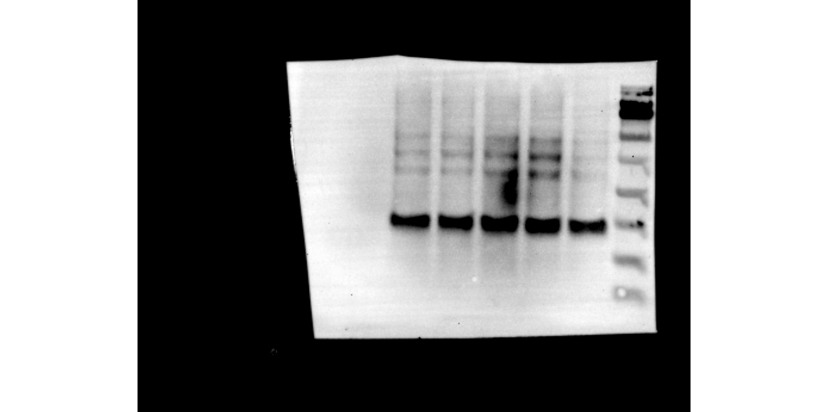
SW837 Total Ras


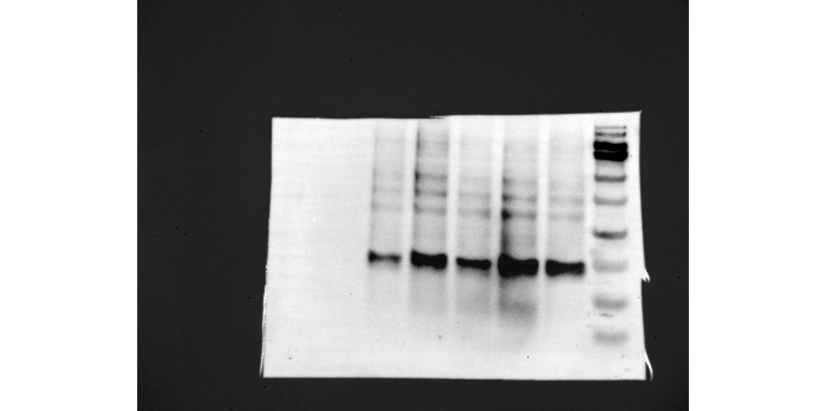
SW837 Ras-GTP


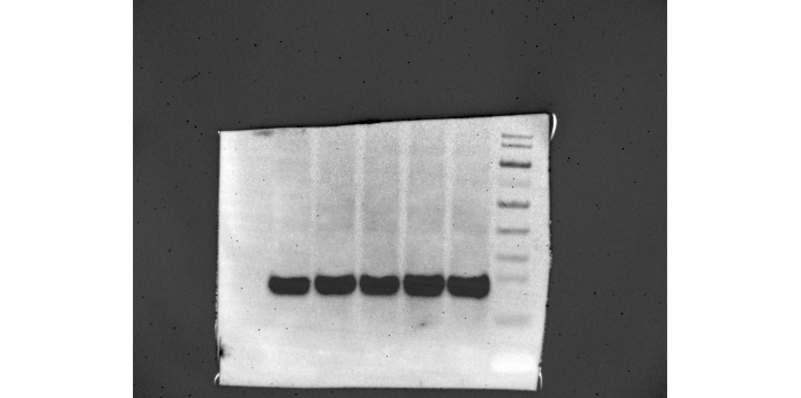
LS180 Total Ras


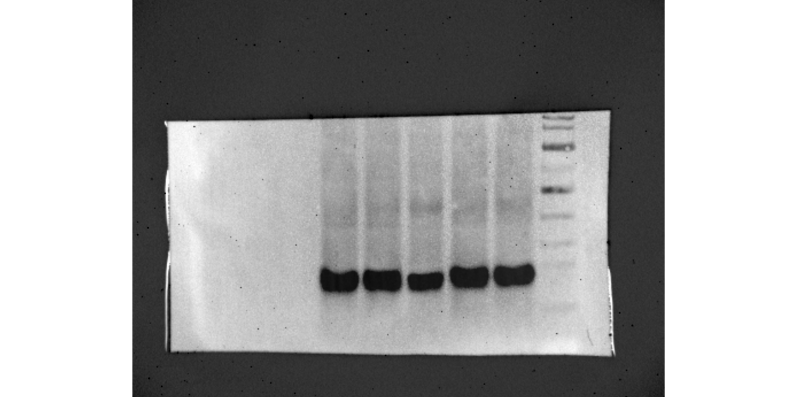
LS180 Ras-GTP


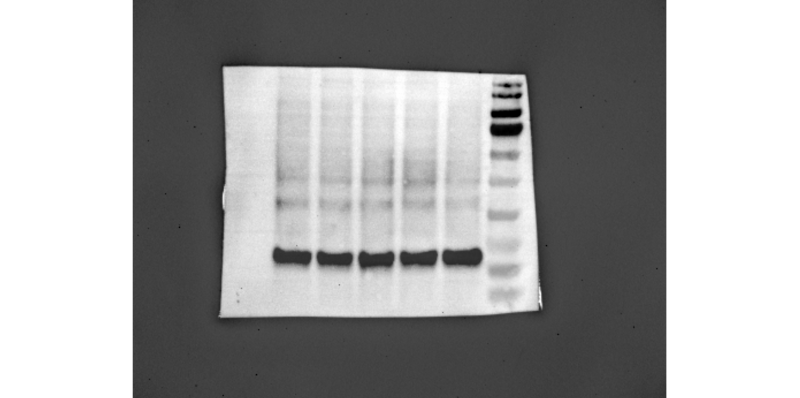
SW480 Total Ras


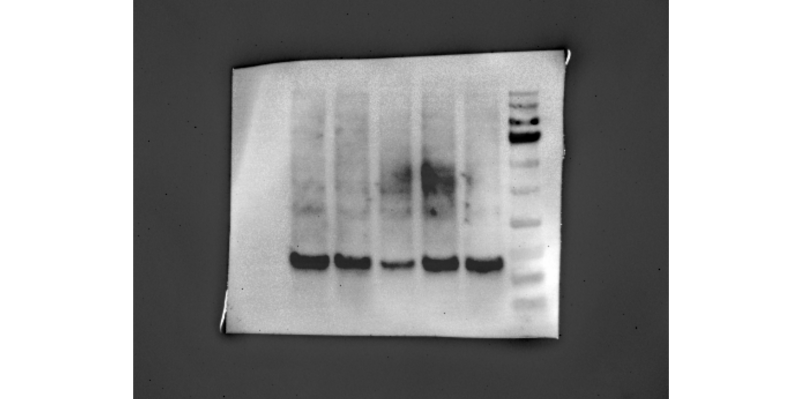
SW480 Ras-GTP


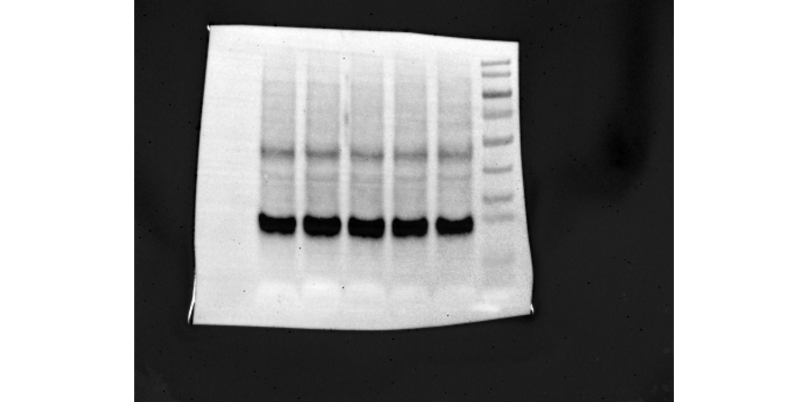
HCT116 Total Ras


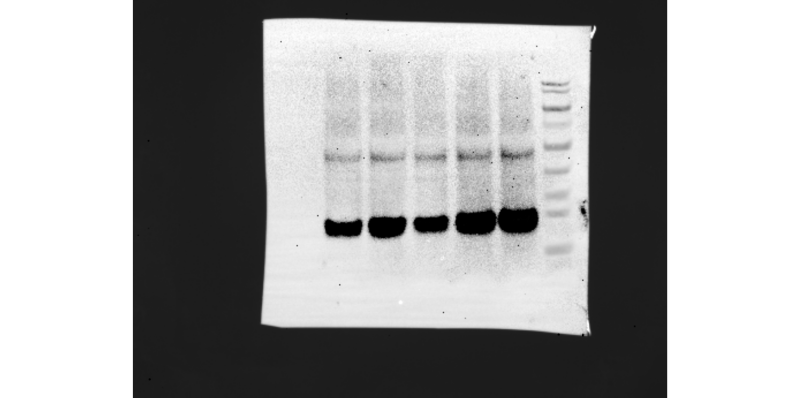
HCT116 Ras-GTP

5B


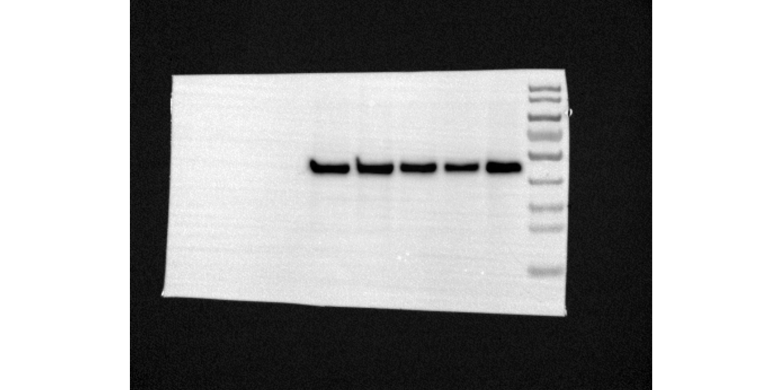
HT29 β-actin


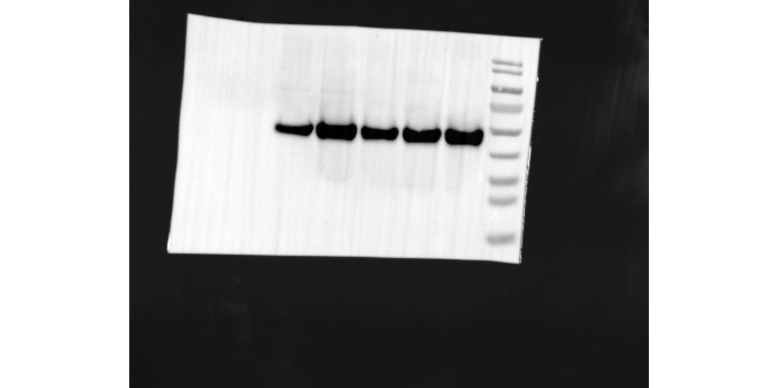
HT29 MEK


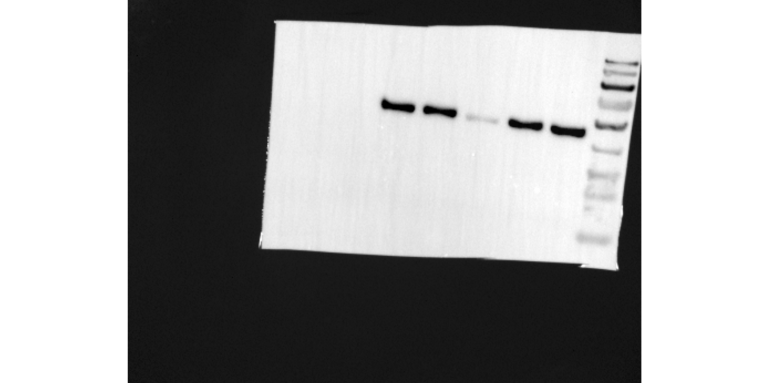
HT29 p-MEK


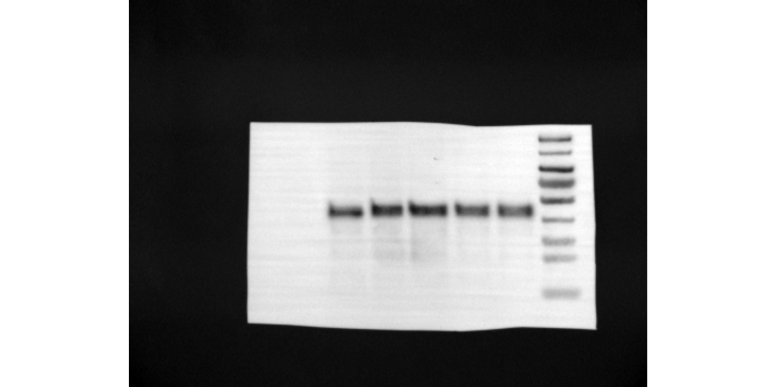
HT29 ERK


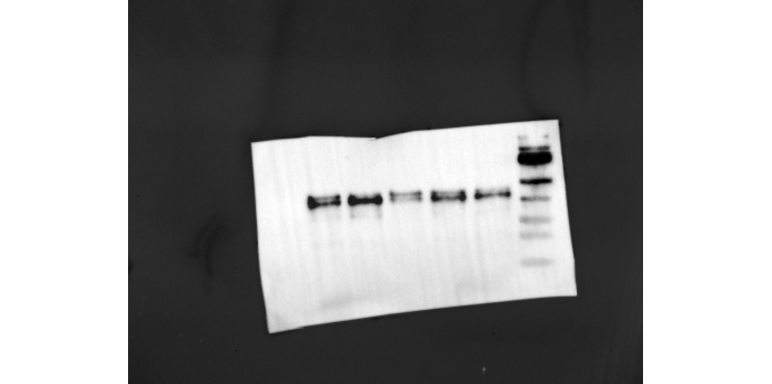
HT29 p-ERK


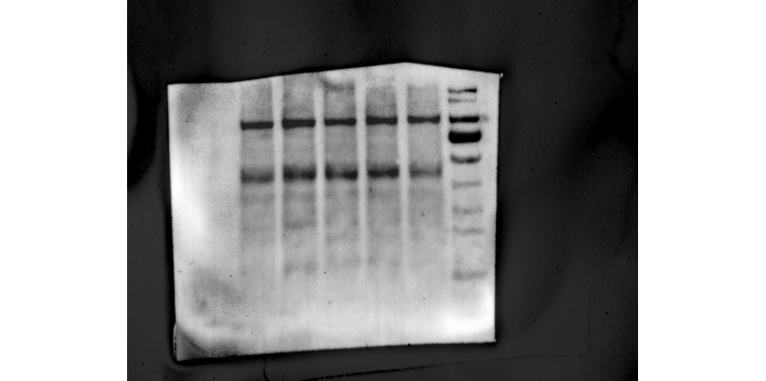
HT29 PI3K


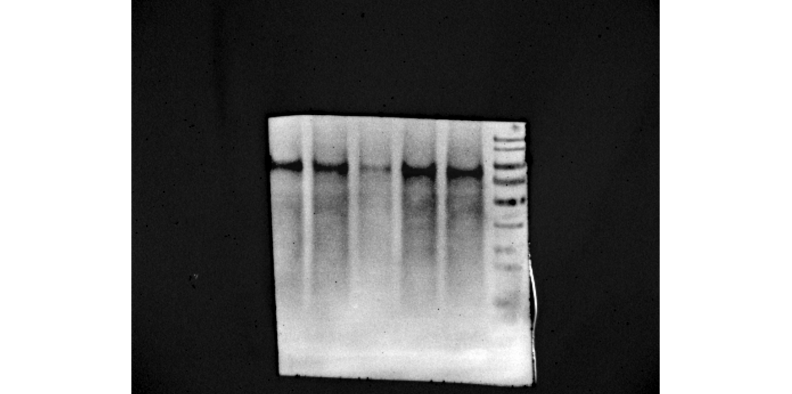
HT29 p-PI3K


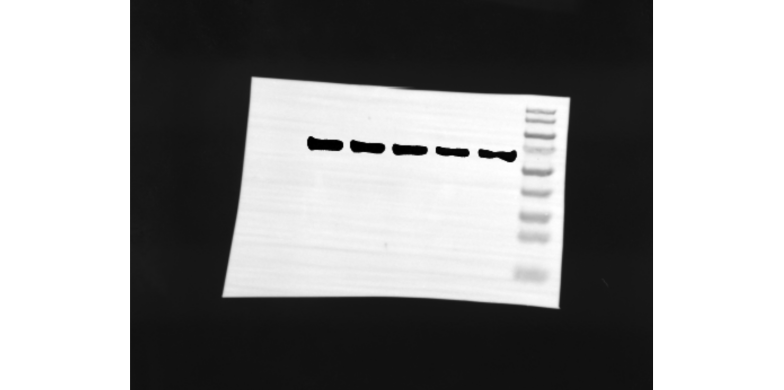
HT29 AKT


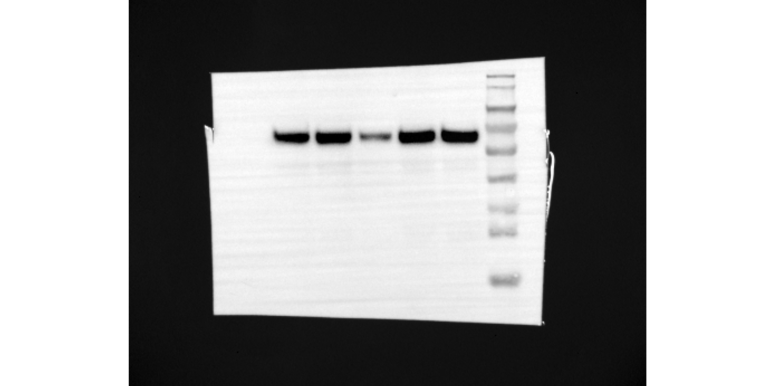
HT29 p-AKT

5C


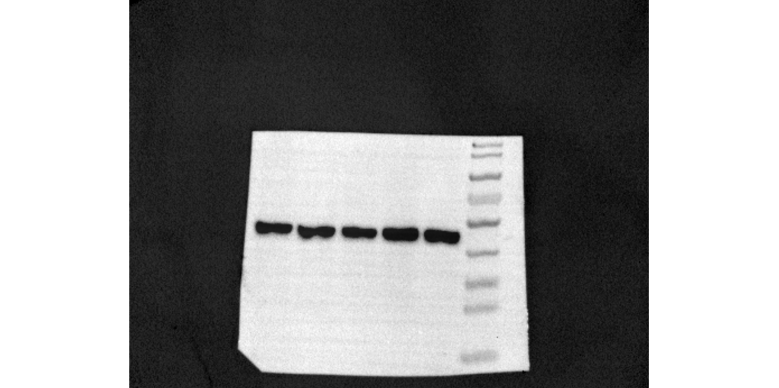
SW837 β-actin


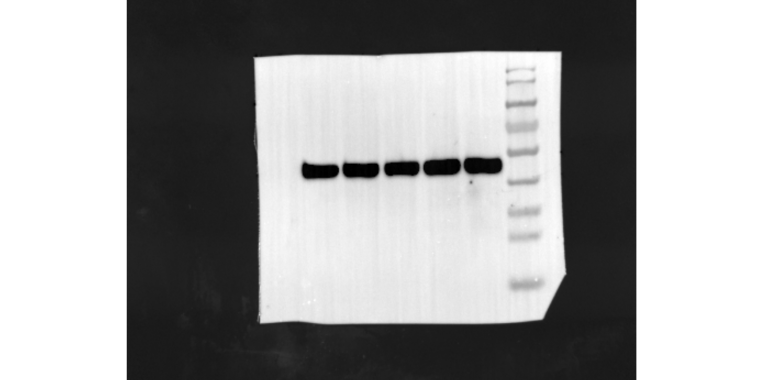
SW837 MEK


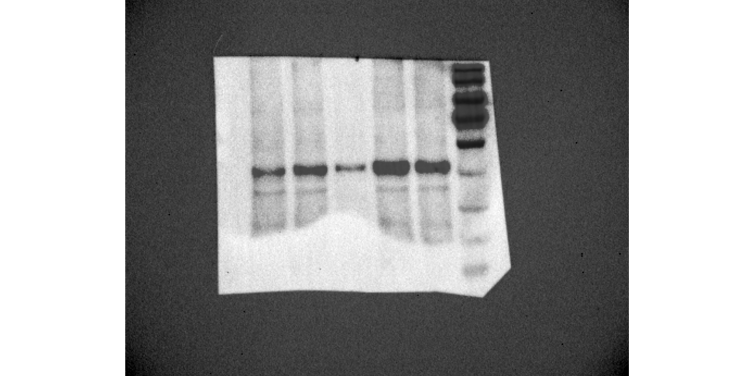
SW837 p-MEK


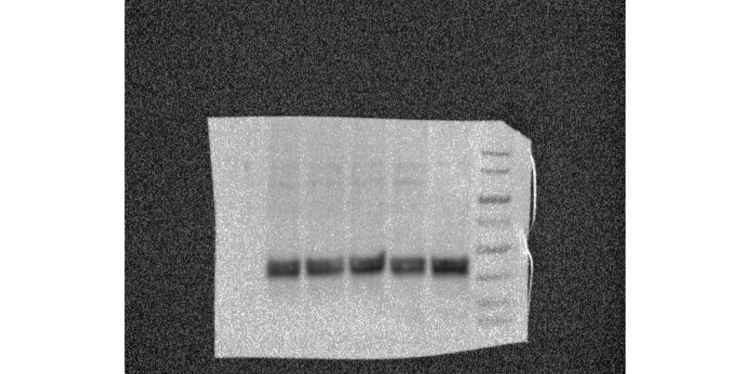
SW837 ERK


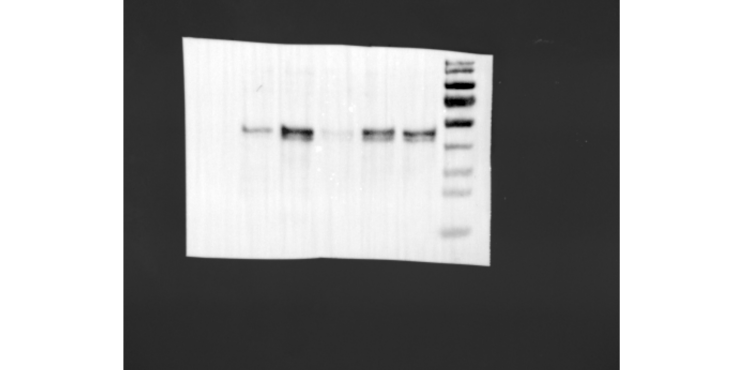
SW837 p-ERK


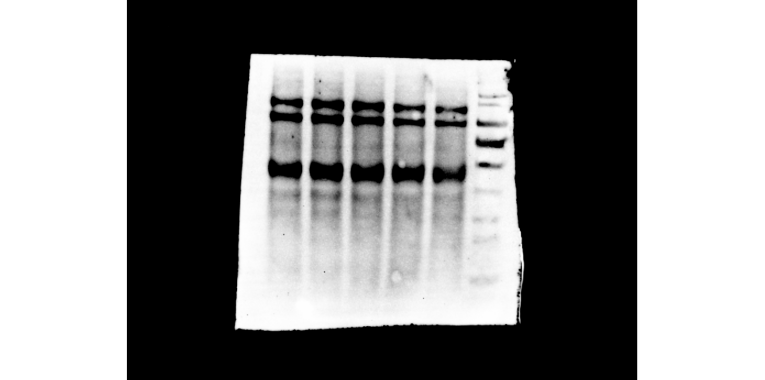
SW837 PI3K


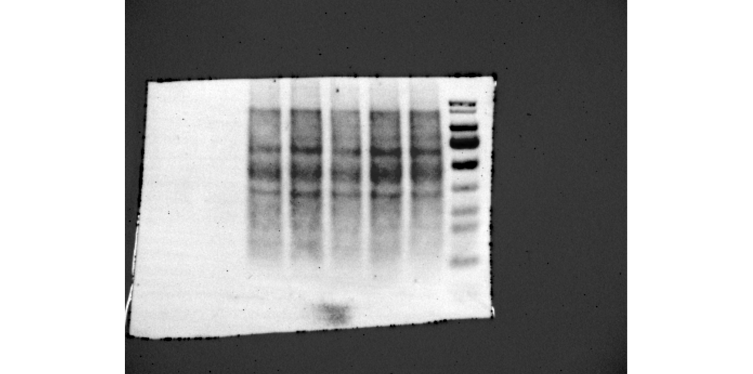
SW837 p-PI3K


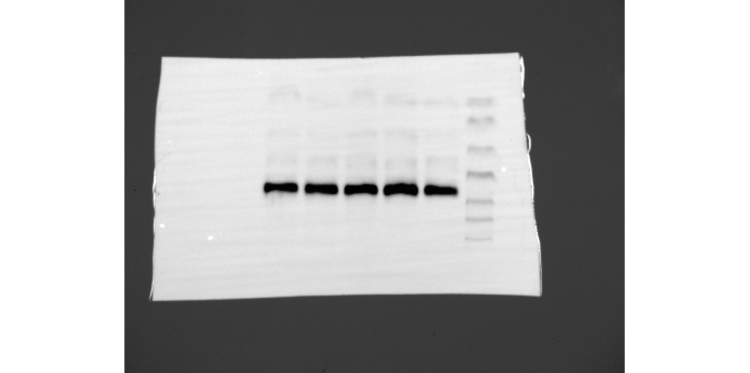
SW837 AKT


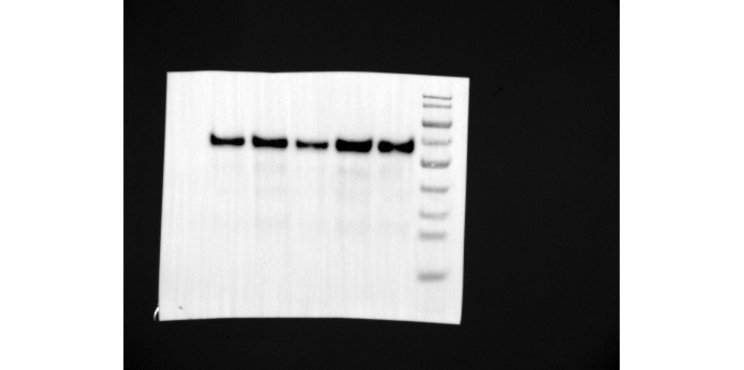
SW837 p-AKT

5D


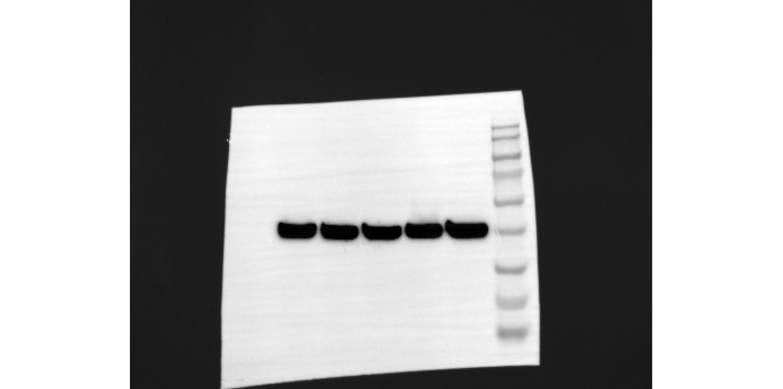
LS180 β-actin


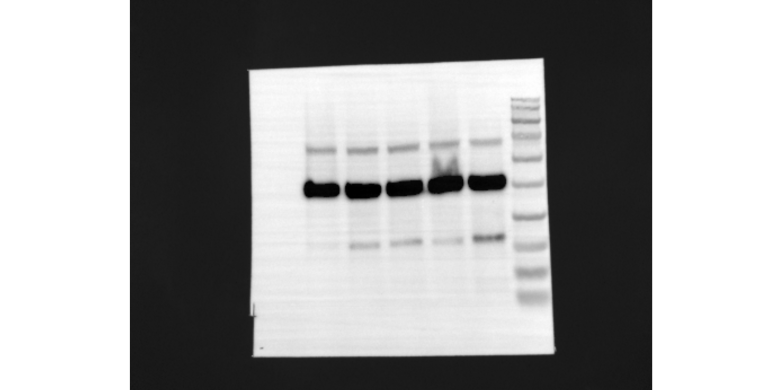
LS180 MEK


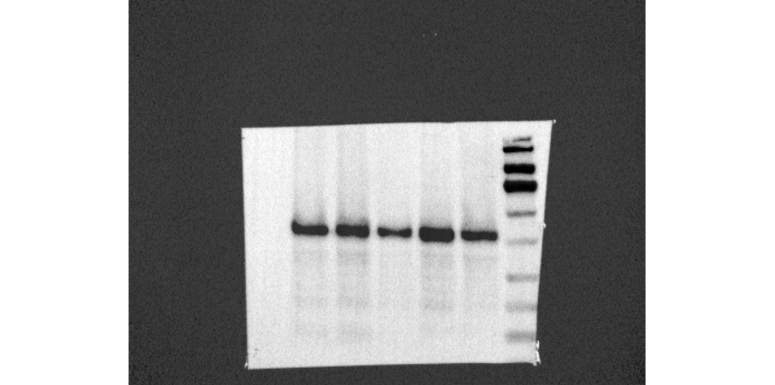
LS180 p-MEK


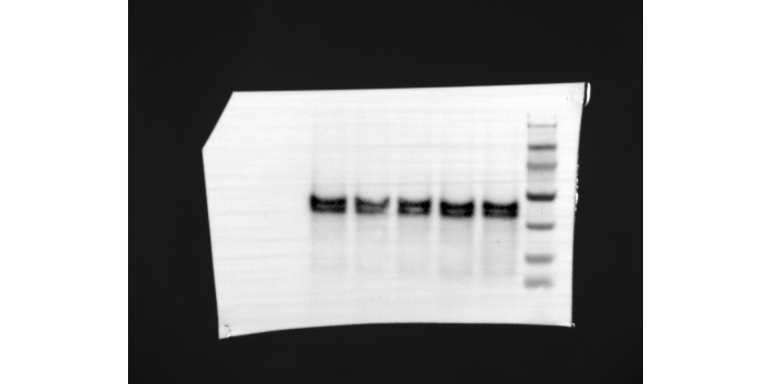
LS180 ERK


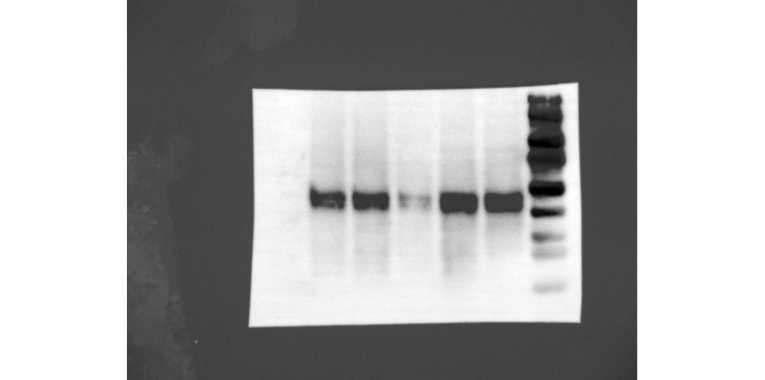
LS180 p-ERK


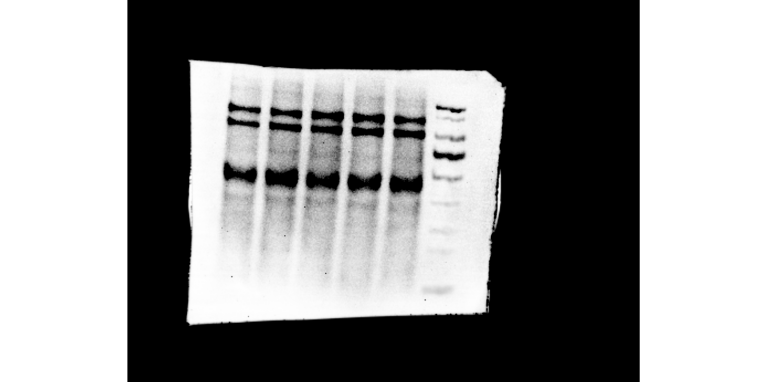
LS180 PI3K


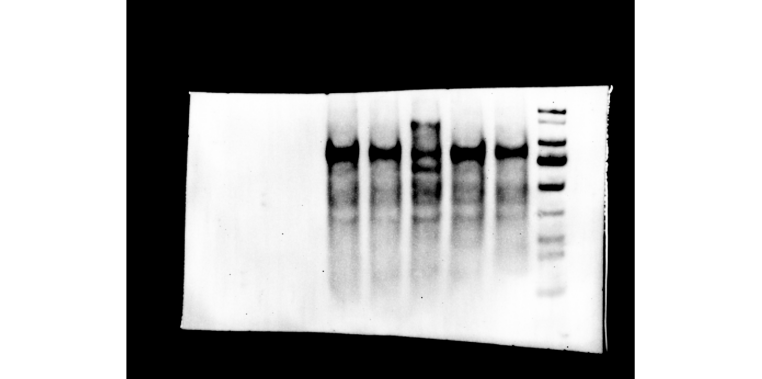
LS180 p-PI3K


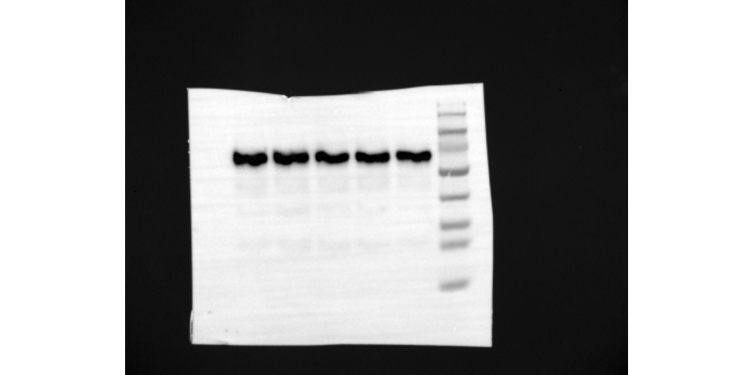
LS180 AKT


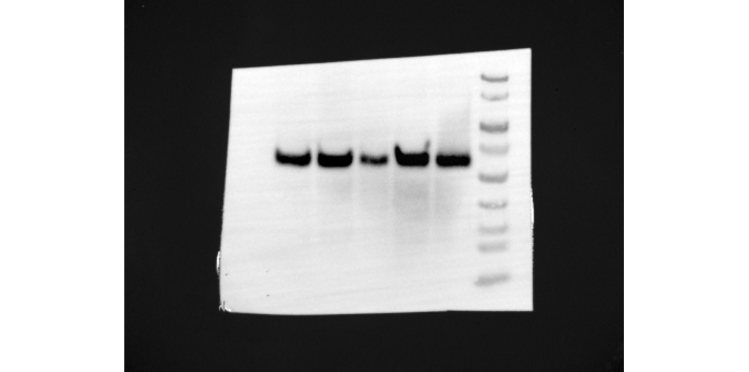
LS180 p-AKT

5E


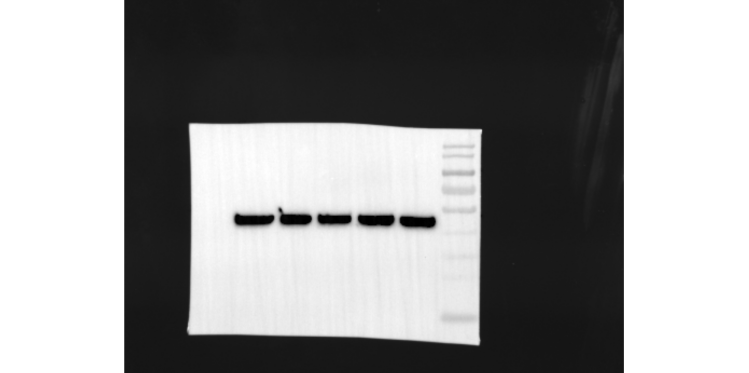
SW480 β-actin


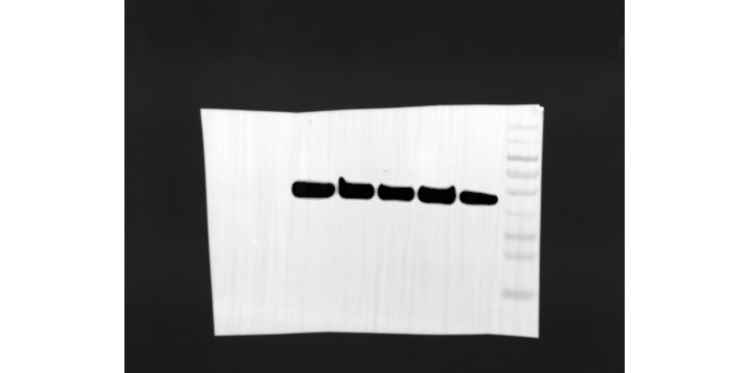
SW480 MEK


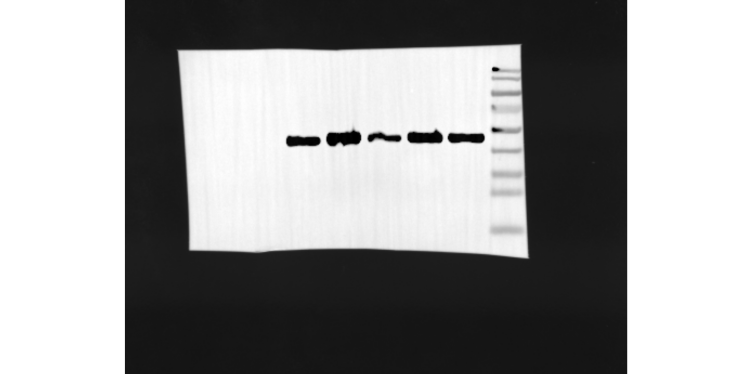
SW480 p-MEK


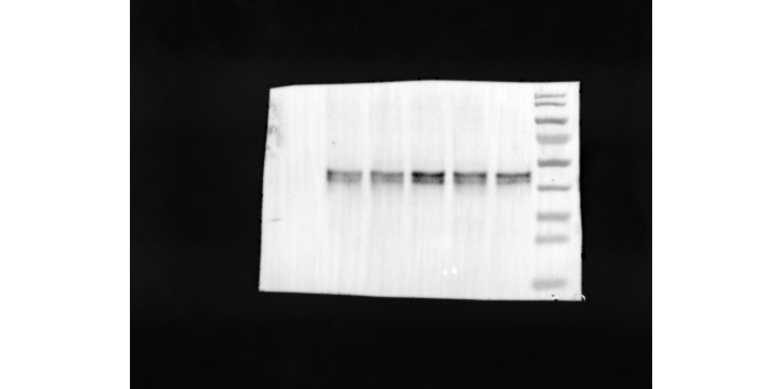
SW480 ERK


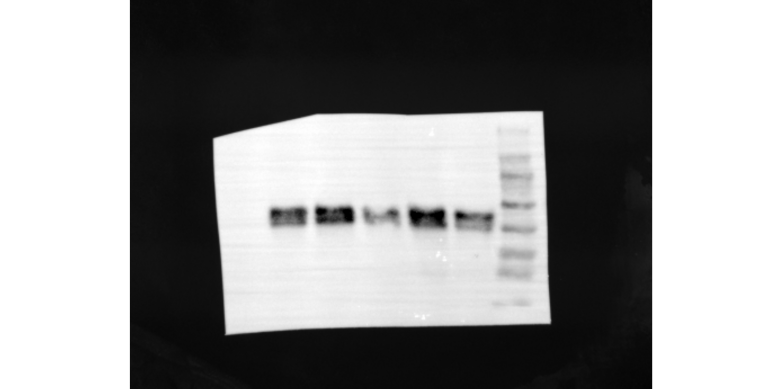
SW480 p-ERK


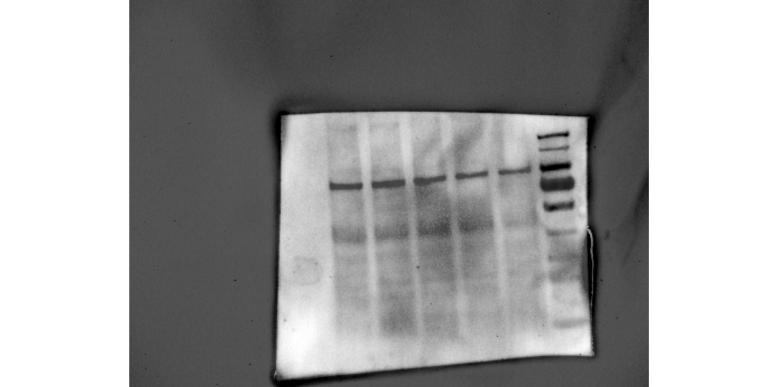
SW480 PI3K


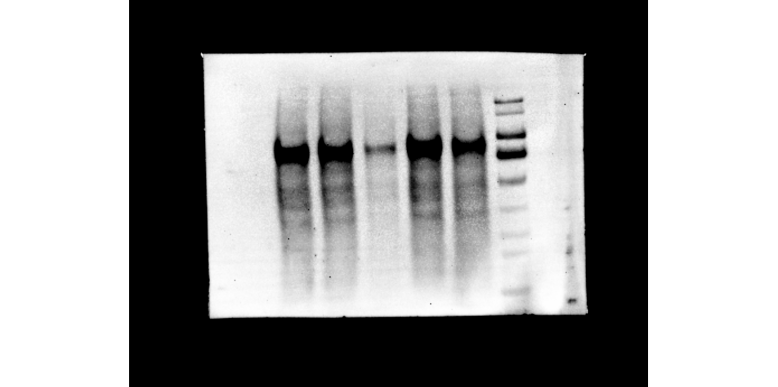
SW480 p-PI3K


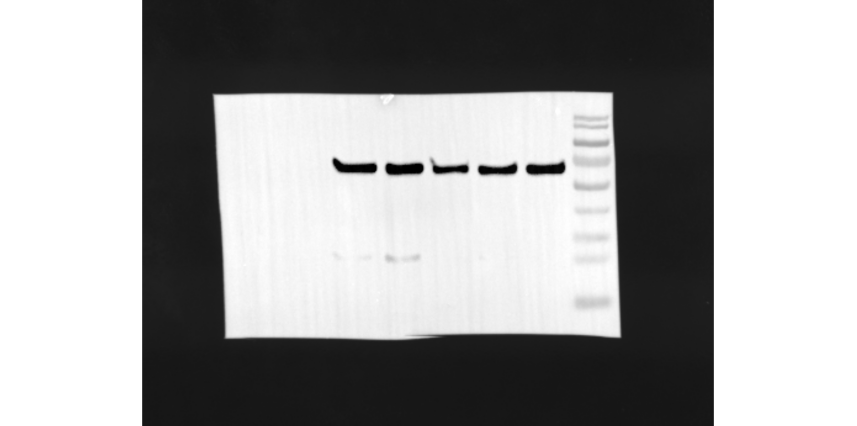
SW480 AKT


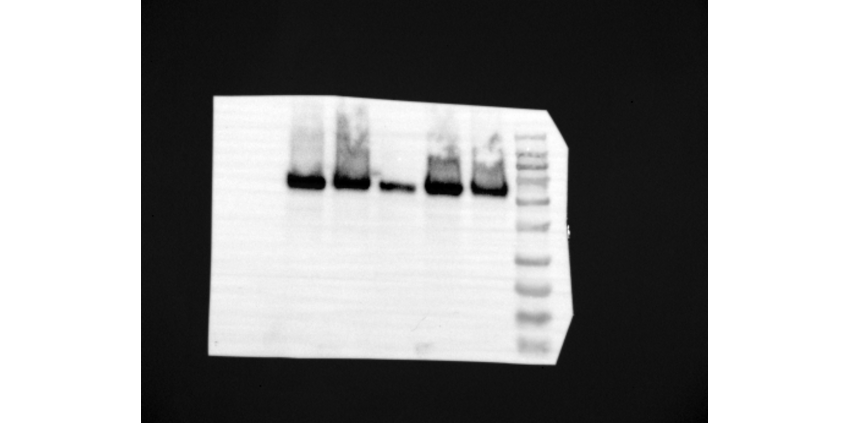
SW480 p-AKT

5F


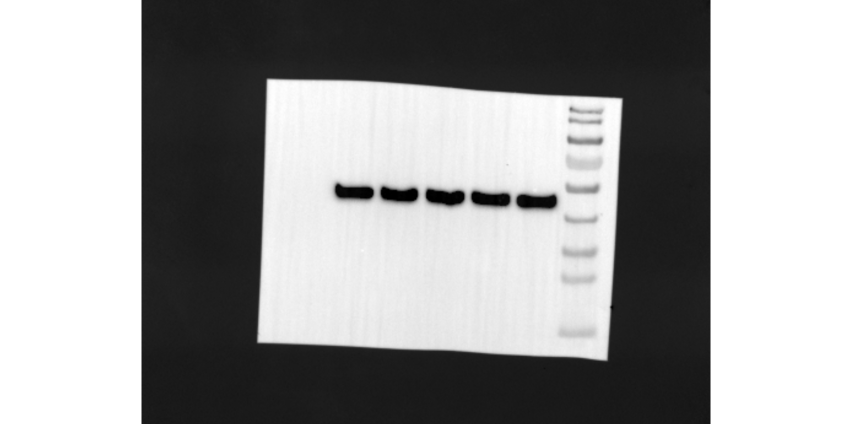
HCT116 β-actin


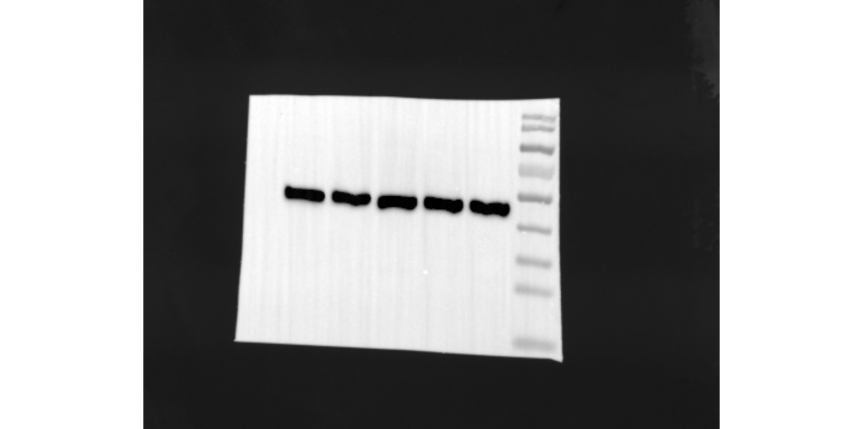
HCT116 MEK


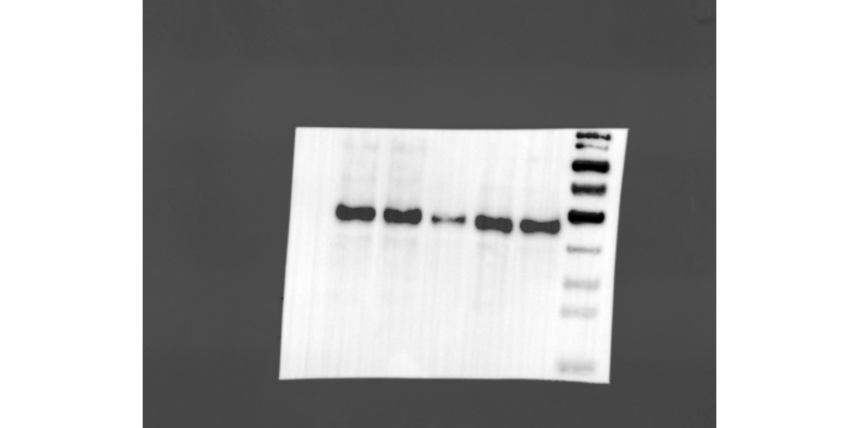
HCT116 p-MEK


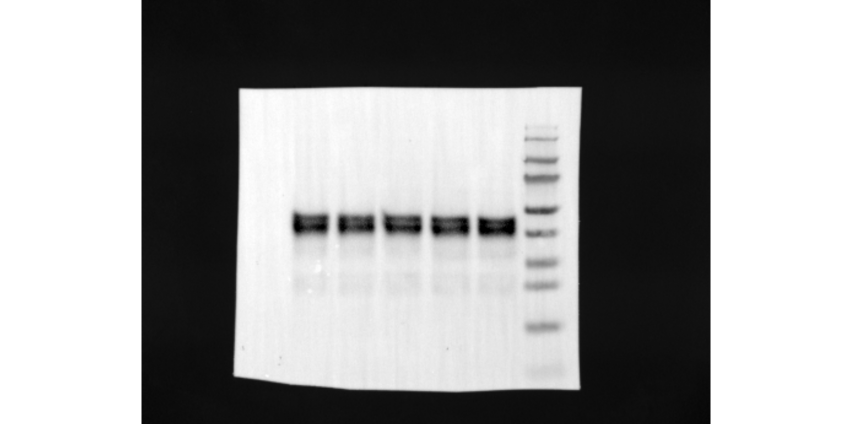
HCT116 ERK


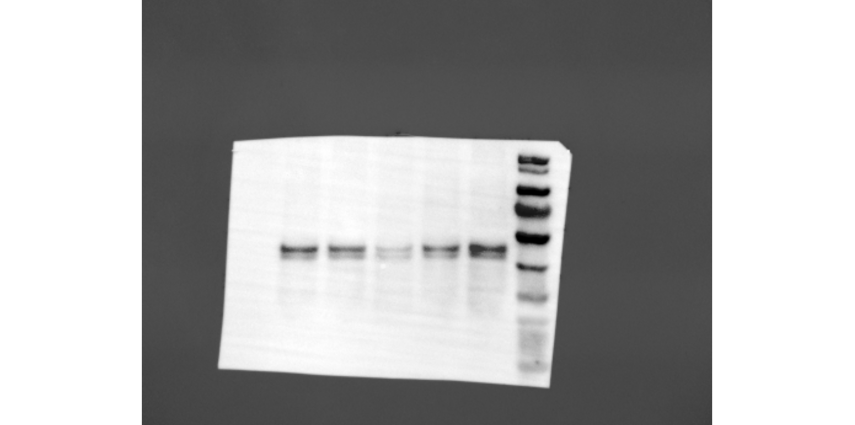
HCT116 p-ERK


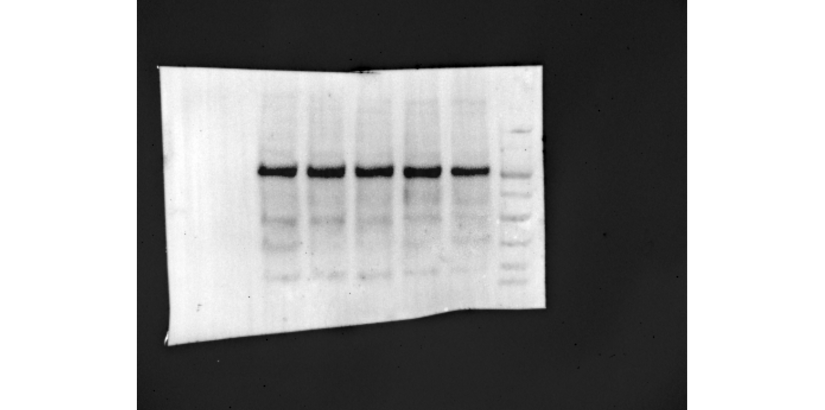
HCT116 PI3K


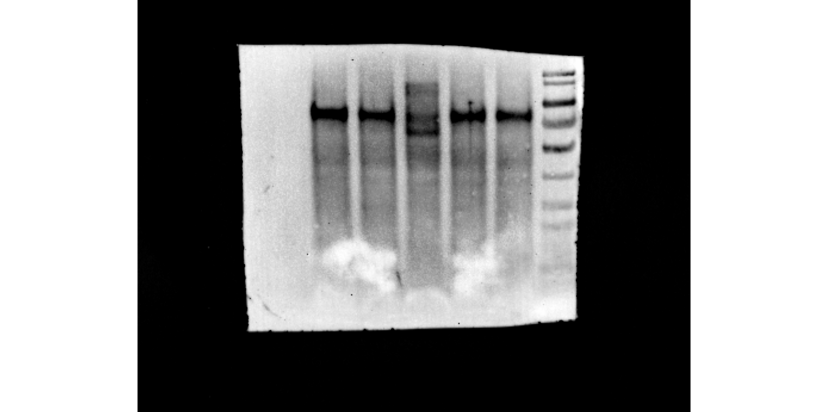
HCT116 p-PI3K


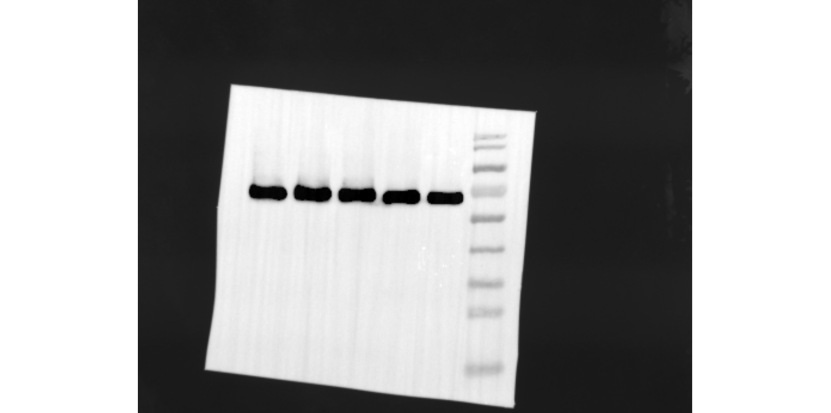
HCT116 AKT


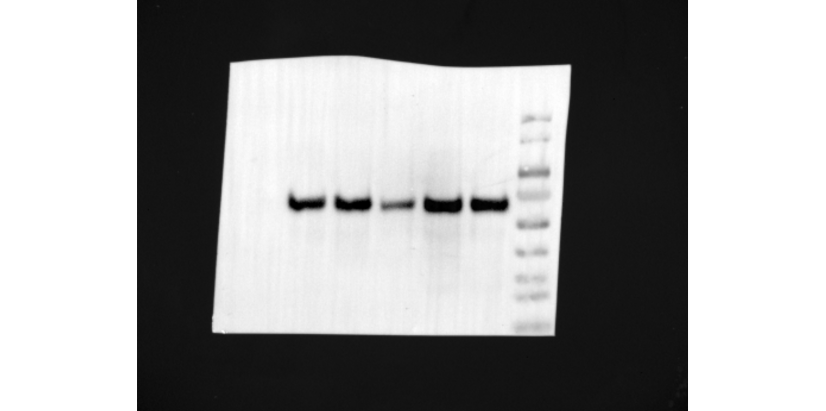
HCT116 p-AKT

S-1C


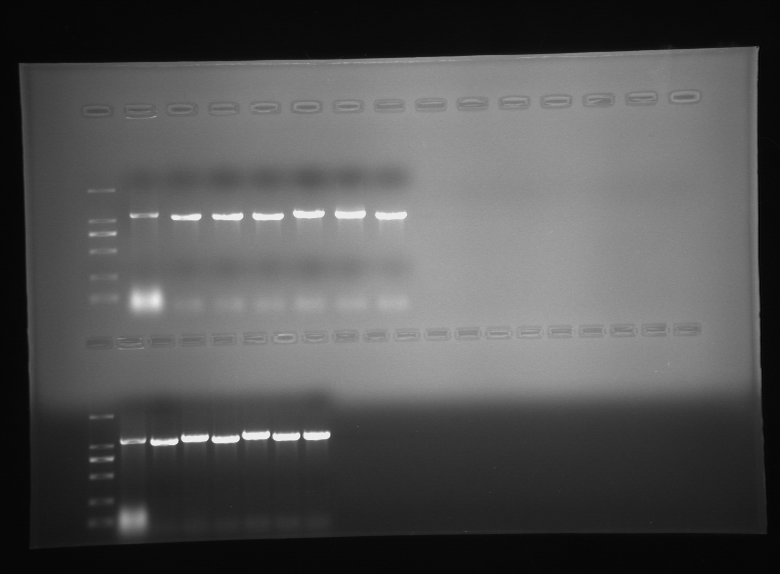


S-1D


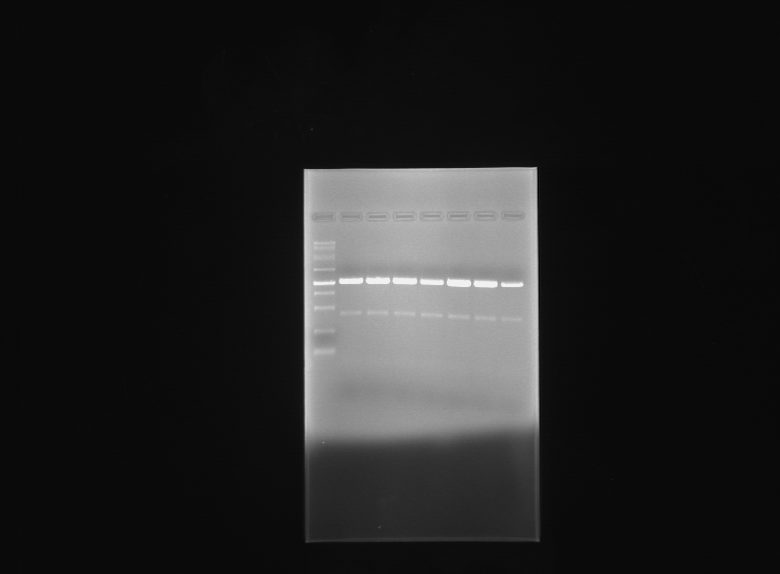


S-1E


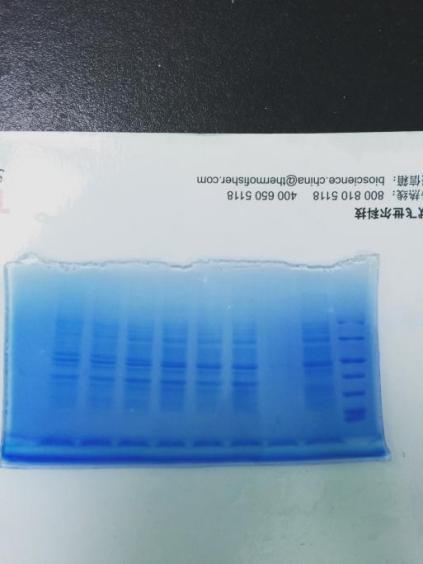


S1F


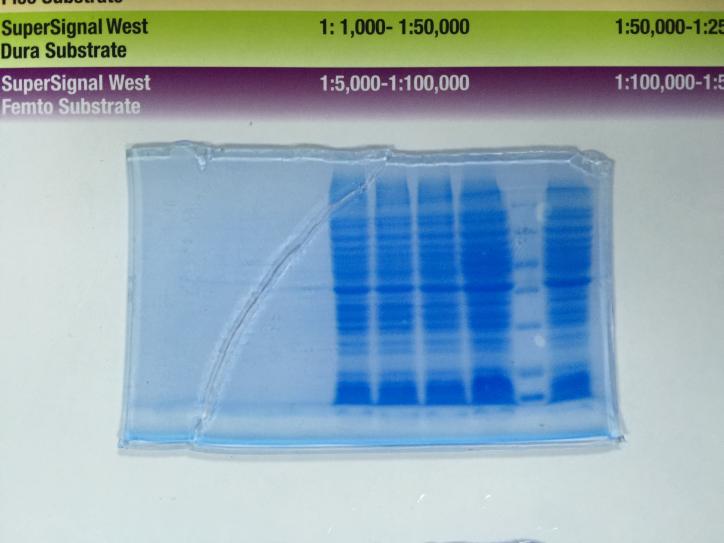


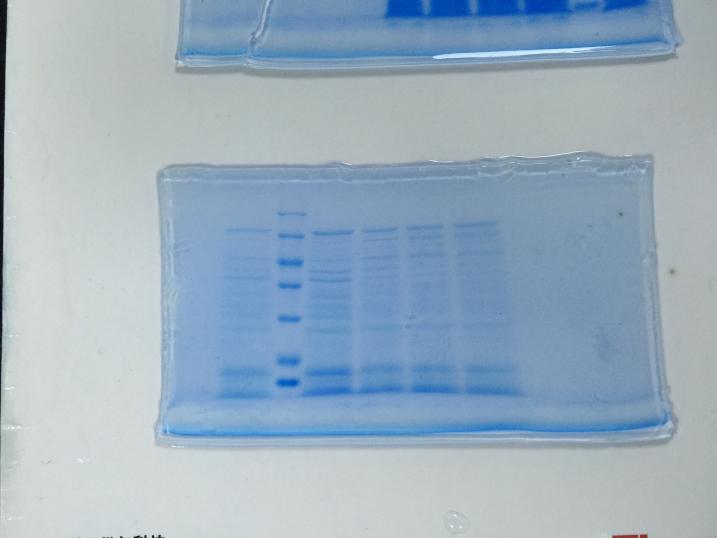


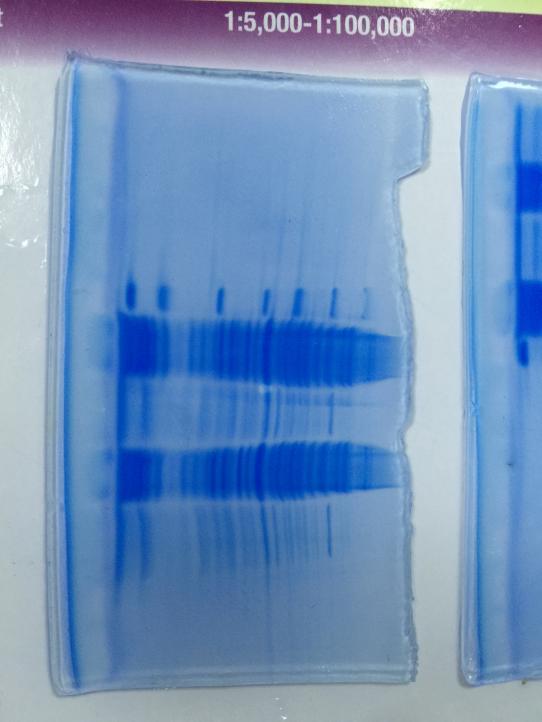


S4


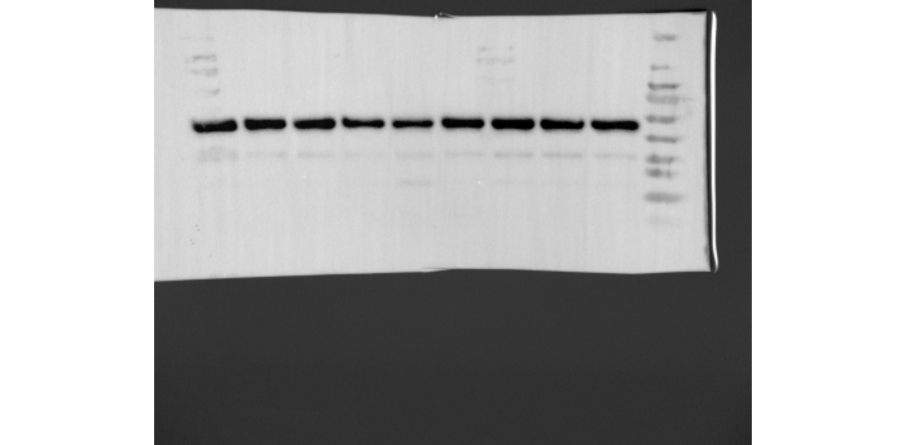
HT29 β-actin


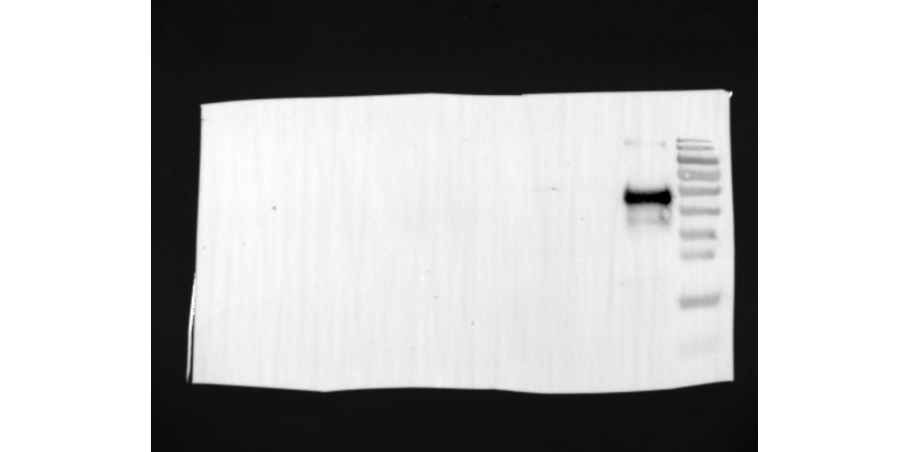
HT29 RGD-p21Ras-scFv


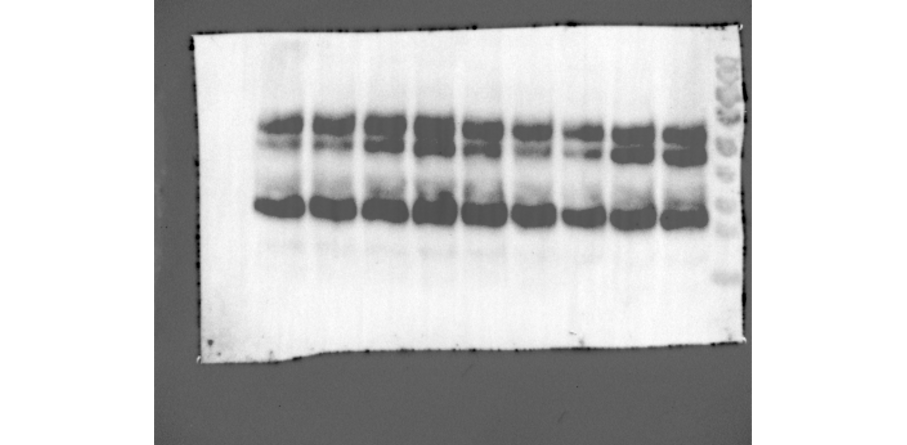
SW480 β-actin


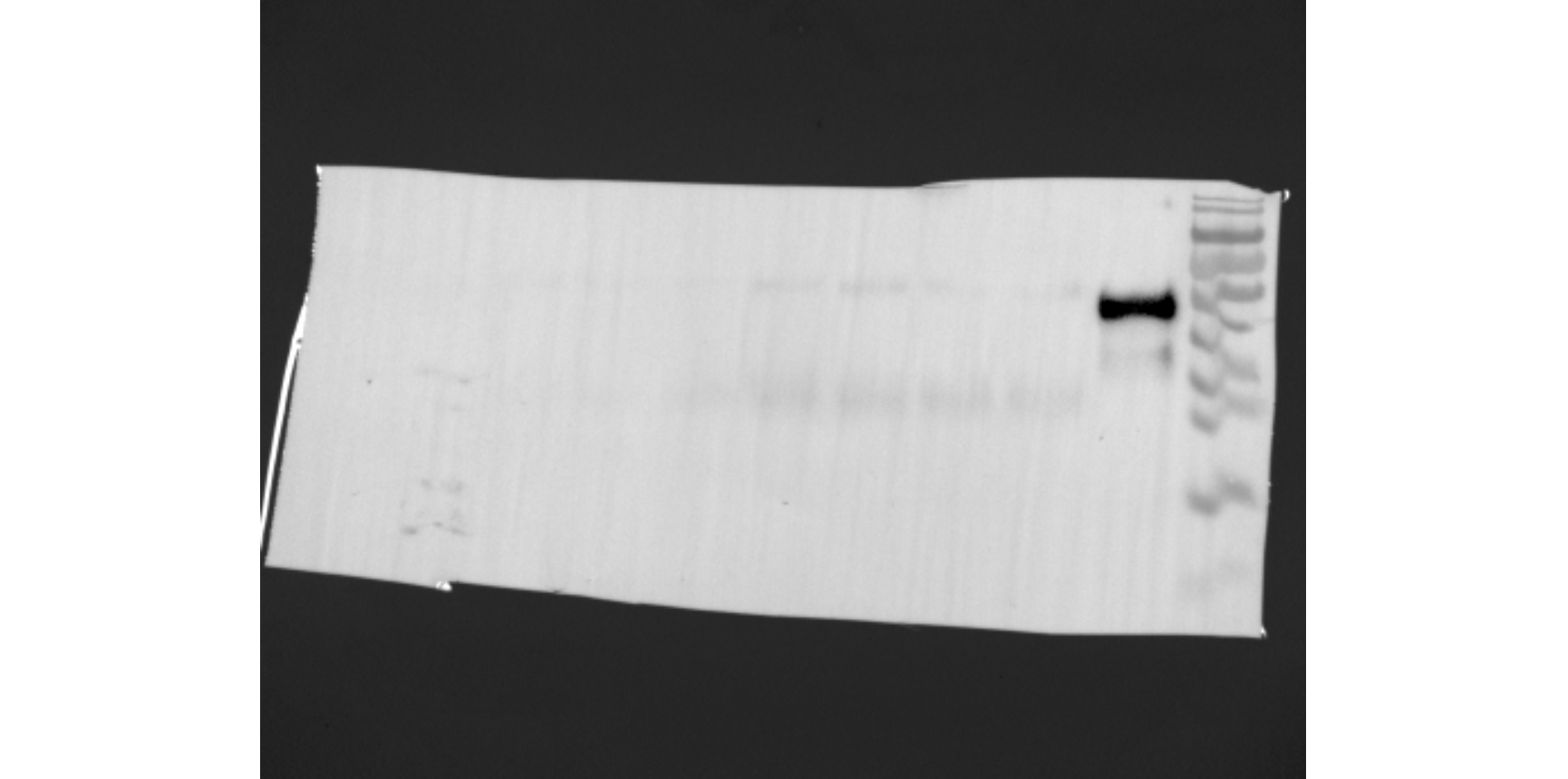
SW480 RGD-p21Ras-scFv
